# From multi-omics to functional validation: the PTMRS stratifies TME and positions PDGFRB in CRC biology

**DOI:** 10.3389/fimmu.2025.1728291

**Published:** 2026-01-12

**Authors:** Yanhong Liu, Chao Lu, Luchun Hua, Hankun Hao, Qijing Zhang, Zongyou Zou, Jianbin Xiang, Yaping Wang

**Affiliations:** 1 Department of General Surgery, Huashan Hospital, Fudan University, Shanghai, China; 2 Department of Physiology and Pathophysiology, School of Basic Medical Sciences, Fudan University, Shanghai, China

**Keywords:** cancer-associated fibroblasts, colorectal cancer, PDGFRB, post-translational modifications, PTMRS, tumor microenvironment

## Abstract

**Background:**

Colorectal cancer (CRC) outcomes remain heterogeneous despite therapeutic advances, posing challenges to precise prognostic stratification. Post-translational modifications (PTMs) critically regulate protein function, tumor microenvironment (TME) crosstalk, and CRC progression, while most existing studies only focus on single PTM types and PTM signals are rarely integrated into CRC risk models.

**Methods:**

We built a post-translational modification–related risk signature (PTMRS) model by screening post-translational modification–related (PTM-related) genes associated with prognosis in The Cancer Genome Atlas (TCGA) and Genotype-Tissue Expression project (GTEx)datasets, then benchmarking 101 modeling strategies to select a Cox partial least squares framework. External validation was conducted across multiple independent cohorts and immunotherapy datasets. Single-cell RNA sequencing (scRNA-seq) data were used to calculate cell type–specific scores for the PTMRS and to perform CellChat-based analyses of cell–cell communication. To investigate mechanism, we targeted platelet-derived growth factor receptor-β (PDGFRβ) in human colonic fibroblasts and assessed CRC-cell responses to their conditioned media.

**Results:**

PTMRS robustly stratified overall survival across cohorts and aligned with an immune-cold, stroma-enriched phenotype. PTMRS was associated with pathways related to antigen presentation, protein homeostasis, and other processes within the CRC TIME. ScRNA-seq analysis further showed that PTMRS scores influenced intercellular communication between CRC cells and immune cells. PDGFRB was validated as a core node within the PTMRS network: activation of PDGFRβ in human colonic fibroblasts promoted CRC cell proliferation and migration, whereas sunitinib attenuated and reversed these effects.

**Conclusions:**

We established a post-translational modification–related risk signature (PTMRS) that robustly stratifies CRC prognosis and links tumor-intrinsic programs with features of the TIME. Higher PTMRS scores, particularly in tumors with *PDGFRB* enrichment, were associated with a stroma-rich, immune-cold phenotype. Experimental validation highlighted PDGFRβ in colonic fibroblasts as a stromal hub whose activation promotes CRC cell proliferation and migration. These findings suggest that PTMRS may help identify patients who could benefit from combining immunotherapy with therapies targeting PDGFRβ or other PTM-related pathways. Further validation in *in vivo* models and prospective clinical cohorts is needed.

## 1 Introduction

Colorectal cancer (CRC) is the third most commonly diagnosed malignancy and the second leading cause of cancer mortality worldwide; its incidence continues to rise, with a growing burden among younger adults (1, 2). Despite advances in surgery, chemotherapy, and immunotherapy, outcomes of CRC patients remain highly variable: the 5-year survival rate is above 90% in early-stage disease but drops to below 15% in metastatic CRC (3). This sharp contrast highlights the urgent need for reliable prognostic biomarkers and clinically relevant therapeutic targets.

Post-translational modifications (PTMs)—including ubiquitination, phosphorylation, acetylation, methylation, and lipidation—govern protein stability, localization, and function, thereby shaping tumor initiation, progression, and metastasis (4, 5). For example, ubiquitination influences tumor growth and apoptosis through degradation of suppressors (e.g., p53) and activation of oncogenic signaling (e.g., phosphatidylinositol 3-kinase [PI3K]-AKT serine kinase [AKT] pathway) (6), while histone methylation and acetylation remodel chromatin and reprogram gene expression (7).However, most CRC studies have focused on individual PTM types or isolated signaling pathways, leaving the broader PTM regulatory network and its interconnected architecture largely unresolved, which in turn limits reproducibility and reduces translational impact.

The tumor microenvironment (TME)—comprising immune cells, stromal elements, and extracellular matrix—profoundly affects tumor behavior and therapeutic response (8). Accumulating evidence shows that TME-resident macrophages and fibroblasts can amplify immunosuppression and drive cancer progression, whereas abundant cytotoxic T-cell infiltration generally predicts favorable prognosis in CRC (9, 10). Although links between PTMs and the TME have begun to emerge, how PTMs reshape the CRC TME and modulate intercellular communication remains unclear.

We hypothesized that integrating signals from multiple PTM-related genes into a composite risk score would more effectively capture stable tumor-biological features across cohorts, thereby improving prognostic assessment, refining immune-phenotype interpretation, and helping to prioritize potential therapeutic targets (11). Accordingly, we combined the TCGA and GTEx datasets to identify PTM-related differentially expressed genes (PTM-related DEGs) that are associated with CRC prognosis. Using a multi-algorithm approach (101 strategies), we further constructed a prognostic model—a post-translational modification-related risk signature (PTMRS). The model was externally validated across multiple independent GEO cohorts and outperformed clinical variables and 22 published signatures. The relationship between PTMRS and the tumor microenvironment (TME) was then investigated by applying cell type–specific PTMRS scoring and CellChat analysis to single-cell RNA sequencing (scRNA-seq) data, thereby mapping immune-microenvironment differences between high-PTMRS and low-PTMRS groups. On this basis, PDGFRB, which encodes platelet-derived growth factor receptor-β (PDGFRβ), a stromal receptor tyrosine kinase implicated in fibroblast activation and angiogenesis, was selected as a potential hub gene. Evidence from expression, survival, immune associations, and pathology was then integrated, and preliminary *in vitro* experiments were performed to evaluate its role in CRC.

This study differs from prior work in three ways. First, rather than centering on a single PTM class (12–14), we surveyed a comprehensive set of PTM-related gene programs to provide a systems-level view of PTM-related regulatory landscape in CRC. Second, benchmarking 101 modeling strategies identified Cox partial least squares (plsRcox) as the final framework for PTMRS, supporting robustness and generalizability. Third, by integrating scRNA-seq, we clarified the distribution of PTMRS across cell types and explored its impact on cell–cell communication. Taken together—from model construction and external validation to targeted experimental analyses—these findings delineate the links between PTMs, the TME, and CRC prognosis. A reliable PTMRS may offer clinicians an efficient tool for risk stratification and outcome prediction, while the identification of hub genes such as PDGFRB points toward new directions for targeted therapy development in advanced CRC.

## 2 Materials and methods

### 2.1 Transcriptomic data acquisition and preprocessing

RNA-seq expression profiles and matched clinical annotations for TCGA-COAD (n = 448) were used to build the prognostic model; independent cohorts served to test model stability and accuracy. Raw counts were converted to transcripts per million (TPM) and log2-transformed for downstream analyses. External validation employed seven GEO microarray datasets—GSE12945 (n = 62), GSE17536 (n = 177), GSE17537 (n = 55), GSE38832 (n = 122), GSE39582 (n = 579), GSE41258 (n = 182), and GSE87211 (n = 196)—yielding a total of 1,821 specimens when combined with TCGA. In addition, seven immunotherapy cohorts were analyzed (sample sizes in parentheses): Melanoma_GSE100797 (25), Melanoma_GSE78220 (28), Melanoma_GSE91061 (109), Melanoma_phs000452 (153), Melanoma_Nathanson_2017 (24), Melanoma_PRJEB23709 (91), and IMvigor210 (348). Differential expression between tumor and non-tumor tissues was computed using TCGA (Normal = 41, Tumor = 471) together with normal colon samples from GTEx (Normal = 308). GEO microarray data were normalized with limma::normalizeBetweenArrays, and cross-study batch effects were mitigated using sva::ComBat.

### 2.2 Acquisition and processing of scRNA-seq data

Single-cell datasets were retrieved from the Gene Expression Omnibus (GEO) database, including GSE231559 (encompassing 6 COAD tumor samples) and GSE200997 (comprising 16 COAD tumor samples) for subsequent analysis. R software (Version 4.1.3) was employed for data analysis, with the Seurat package selected as the core tool for scRNA-seq data processing.

For cell quality control (QC), the following criteria were applied: mitochondrial content was required to be less than 20% (25% for GSE200997), hemoglobin content was restricted to less than 3%, and the number of unique molecular identifiers (UMIs) and detected genes per cell were constrained within the ranges of 200–25,000 and 200–7,000, respectively (adjusted to 200–25,000 and 200–4,000 for GSE200997).

Data normalization, selection of highly variable genes (top 2,000 genes), and data scaling (with cell cycle effects eliminated using the parameter vars.to.regress = c(“S.Score”, “G2M.Score”)) were performed using the NormalizeData, FindVariableFeatures, and ScaleData functions from the Seurat package, respectively. Batch effects were corrected using the Harmony algorithm. For downstream dimensionality reduction and clustering, the Uniform Manifold Approximation and Projection (UMAP) method and Louvain clustering algorithm—both integrated within the Seurat package—were utilized. The FindAllMarkers function was used to identify differentially expressed genes (DEGs) between cell clusters or cell types, with filtering parameters set as follows: p-value < 0.05, log2 fold change (log2FC) > 0.25, and gene expression proportion > 0.1.

### 2.3 Cell annotation analysis

Cell annotation was initially conducted using canonical cell-type-specific markers: epithelial cell markers: “EPCAM”, “KRT18”, “KRT19”, “CDH1”; Fibroblast markers: “DCN”, “THY1”, “COL1A1”, “COL1A2”; Endothelial cell markers: “PECAM1”, “CLDN5”, “FLT1”, “RAMP2”; T cell markers: “CD3D”, “CD3E”, “CD3G”, “TRAC”; Natural killer (NK) cell markers: “NKG7”, “GNLY”, “NCAM1”, “KLRD1”; B cell markers: “CD79A”, “IGHM”, “IGHG3”, “IGHA2”; Plasma cell marker: “JCHAIN”; Myeloid cell markers: “LYZ”, “MARCO”, “CD68”, “FCGR3A”; Mast cell markers: “KIT”, “MS4A2”, “GATA2”. Based on the annotation results, visualization plots—including UMAP projections, bar charts, and heatmaps—were generated to illustrate cell type distribution and marker gene expression patterns.

### 2.4 Acquisition of prognostic genes

The limma package was used to identify DEGs between tumor and adjacent normal tissues, with the threshold set as |log fold change (logFC)| > 0.6 and p-value < 0.05. From these DEGs, those associated with PTMs were filtered out, referring to the PTM-related gene list described in the study (15). Subsequently, univariate Cox regression analysis was performed to screen for PTM-related DEGs with prognostic significance (P < 0.05). A total of 30 such prognostic genes were ultimately selected as the core gene set for constructing the PTMRS model.

### 2.5 Construction of a tumor-related risk signature

We assembled prognostic models using 101 machine-learning strategies and generated an individual risk score for each patient accordingly. The optimal dichotomization threshold was determined with “surv_cutpoint”, after which patients in TCGA and all validation cohorts were divided into high- and low-risk groups. We then compared survival predictions between groups and quantified overall model accuracy.

### 2.6 Risk signature generated by ensemble machine learning approaches

To develop the PTMRS model with high accuracy and stability, an ensemble approach integrating 10 individual machine learning algorithms and 101 algorithm combinations was adopted. The included algorithms were: Random Survival Forest (RSF), Elastic Net (Enet), Lasso regression, Ridge regression, Stepwise Cox regression, CoxBoost, Cox Partial Least Squares Regression (plsRcox), Supervised Principal Component Analysis (SuperPC), Gradient Boosting Machine (GBM), and Survival Support Vector Machine (survival-SVM). Signature construction proceeded as follows: First, univariate Cox regression was conducted on the integrated dataset (including TCGA data) to identify prognostic genes, as detailed in the previous step; next, 101 algorithm combinations were applied to these prognostic genes to fit predictive models via Leave-One-Out Cross-Validation (LOOCV) in the TCGA-COAD cohort; subsequently, all models were validated in independent validation datasets; finally, Harrell’s Concordance Index (C-index) was calculated for each model across validation datasets, and the model with the highest average C-index was selected as the optimal PTMRS.

### 2.7 Intercellular communication analysis

The CellChat package was used to evaluate potential intercellular communication networks. First, the normalized gene expression matrix was imported using the CellChat function to create a CellChat object. Data preprocessing was then conducted using the identifyOverExpressedGenes, identifyOverExpressedInteraction, and “ProjectData functions with default parameters. Subsequently, the computeCommunProb, filterCommunication, and computeCommunProbPathway functions were applied to identify potential ligand-receptor interactions. Finally, the aggregateNet function was used to construct and visualize the intercellular communication network.

### 2.8 Somatic alteration profiling

Copy-number variations (CNVs) were analyzed with GISTIC2.0, and tumor mutational burden (TMB) was computed using the maftools R package.

### 2.9 Association between the prognostic model and tumor immunity

Immune infiltration in TCGA samples was quantified using IOBR, which aggregates outputs from seven deconvolution/estimation tools. The resulting scores were visualized as heatmaps to compare relative immune-cell proportions within the TME across risk groups.

### 2.10 Drug sensitivity analysis

Drug response was inferred with oncoPredict, estimating IC50 and AUC values from GDSCv2 and CTRP references. Associations between the risk score and drug sensitivity were tested, and Wilcoxon rank-sum tests were used to compare IC50 or AUC between high- and low-risk groups.

### 2.11 Prediction of immunotherapy response

The TIDE framework (Tumor Immune Dysfunction and Exclusion) was applied to TCGA data to estimate immune-response likelihood and to compare predicted immunotherapy benefit between risk strata.

### 2.12 Cell culture

Human CRC cell line HCT116 and colonic fibroblast cell line (CCD-18Co) were obtained from the American Type Culture Collection (ATCC) and cultured in Dulbecco’s Modified Eagle Medium (DMEM) supplemented with 10% fetal bovine serum (FBS) and 1% penicillin-streptomycin at 37 °C in a 5% CO_2_ humidified incubator. To prepare conditioned medium (CM) for downstream assays, CCD-18Co fibroblasts at 70–80% confluence were changed to basal medium with 1% FBS and serum-starved for 12 h, then assigned to three conditions: vehicle (0.1% DMSO), the BB homodimer of platelet-derived growth factor (PDGF-BB, 30 ng/mL), or PDGF-BB plus sunitinib (5 μM). For acute phosphorylation readouts, treatments lasted 15–30 min; for CM production, stimulation continued for 24 h. To limit reagent carryover, cultures were rinsed twice with PBS, replaced with drug-free, low-serum medium (2% FBS), and incubated a further 24 h before supernatants were collected. CM was cleared by centrifugation (300 × g, 5 min), filtered (0.22 μm), and stored at 4 °C (≤48 h) or -80 °C.

### 2.13 Western blot analysis

CCD-18Co cells from the three treatment groups were lysed with RIPA lysis buffer (containing 1% protease inhibitor cocktail and 1% phosphatase inhibitor cocktail) on ice for 30 minutes. Proteins were separated on 4–12% Bis-Tris gradient gels (15-well, M41215C) using 1× MOPS-SDS running buffer at a constant 150 V for 35min and transferred onto a PVDF membrane (Millipore). Membranes were blocked in 5% nonfat dry milk at room temperature (RT) for 1 h, incubated with the indicated primary antibody overnight at 4 °C, and then with the appropriate secondary antibody for 1 h at RT. Signals were developed using enhanced chemiluminescence (ECL; Bio-Rad) and captured on a Tanon gel documentation system. The antibody used for western blot analysis was PDGFR beta (1:2000, Huaan, T1605-20), Phospho-PDGFR beta (Y751) (1:5000, Huaan, HA723550), and β-actin (1:100000, Proteintech, 66009-1-Ig).

### 2.14 Cell counting kit-8 assay for cell viability

HCT116 cells were seeded at 4,000 cells/well in 96-well plates. After attachment, cells were serum-starved for 12 h, after which the medium was replaced with conditioned medium (Veh CM, PDGF-BB CM, or SUN CM) collected from serum-starved CCD-18Co cultures, with six technical replicates per condition. After 48 h of treatment, 10 µL of CCK-8 reagent (Beyotime, C0038, China) was added per well, and the plates were incubated for 1 h at 37 °C. Absorbance at 450 nm was then recorded on a microplate reader. All experiments were performed in ≥3 independent biological replicates.

### 2.15 EdU assay for cell proliferation

HCT116 cells were seeded onto sterile glass coverslips placed in 24-well plates at 5 × 10^4^ cells/well and allowed to adhere for 24 h. After attachment, cells were serum-starved for 12 h, then the medium was replaced with 500 μL conditioned medium (Veh CM, PDGF-BB CM, or SUN CM; n = 3 wells/condition) and incubated for a further 48 h. EdU incorporation and nuclear counterstaining were performed according to the manufacturer’s instructions for the BeyoClick EdU Cell Proliferation Kit with AF488 (Beyotime, C0071L). Fluorescence images were captured at 10× magnification, and five random fields per well were analyzed in ImageJ to enumerate EdU-positive (green) and DAPI-positive (blue) cells.

### 2.16 Transwell assay for cell migration

HCT116 cells were detached with trypsin, washed, and resuspended in serum-free DMEM at 5 × 10^5^ cells/mL. 200 µL of the cell suspension was added to the upper chamber of Transwell inserts (8-µm pores), and 600 µL of conditioned medium (Veh CM, PDGF-BB CM, or SUN CM; n = 3 inserts per group) was placed in the lower chamber. Plates were incubated for 24 h at 37 °C, 5% CO_2_. After incubation, cells remaining on the top side of the membrane were gently wiped off with a cotton swab. Cells on the underside were fixed in 4% paraformaldehyde for 15 min, stained with 0.1% crystal violet for 10 min, and washed three times with PBS. Images were taken with an inverted microscope at 10×, and migrated cells were counted in ImageJ to obtain migrated cell numbers.

### 2.17 Enzyme-linked immunosorbent assay for fibroblast-derived cytokines

Concentrations of C-C motif chemokine ligand 5 (CCL5), C-X-C motif chemokine ligand 12 (CXCL12), interleukin-6 (IL-6), interleukin-10 (IL-10), and transforming growth factor-β1 (TGF-β1) were measured in the conditioned media(CM) of human colonic fibroblasts CCD-18Co (processed, collected as described above, and stored at −80 °C) using human enzyme-linked immunosorbent assay (ELISA) kits from R&D Systems (CCL5, Catalog #DRN00B; CXCL12, Catalog #DSA00; IL-6, Catalog #D6050B; IL-10, Catalog #D1000B; TGF-β1, Catalog #DB100C).Recombinant cytokine standards provided with each kit were serially diluted to generate standard curves. A total of 100 μL of standards and samples (run in six replicates) were added to pre-coated 96-well plates, followed by incubation at room temperature in accordance with the manufacturer’s instructions. Subsequent steps included washing the plates, incubating with horseradish peroxidase-conjugated detection antibodies, performing a second round of washing, and adding substrate solution. The reaction was terminated by adding stop solution, and the absorbance was read at 450 nm using a microplate reader. Cytokine concentrations were calculated from the standard curves and expressed as pg/mL.

### 2.18 Statistical analysis

All database-sourced preprocessing, statistics, and graphics were carried out in R 4.1.3. Associations between continuous measures were examined using Pearson’s correlation; chi-square tests were used for categorical comparisons, and Wilcoxon rank-sum tests for between-group differences in continuous variables. The survminer package was used to determine optimal cut points, and survival analyses (Cox proportional hazards and Kaplan–Meier curves) were performed with the survival package. Experimental validation data were analyzed in Prism 9.4.1 (GraphPad Software, La Jolla, CA) Two-sample, two-tailed *t*-tests were applied for two-group comparisons. Statistical significance was set at P < 0.05.

## 3 Results

### 3.1 Screening and characterization of PTM-related DEGs in CRC

**Figure 1:**
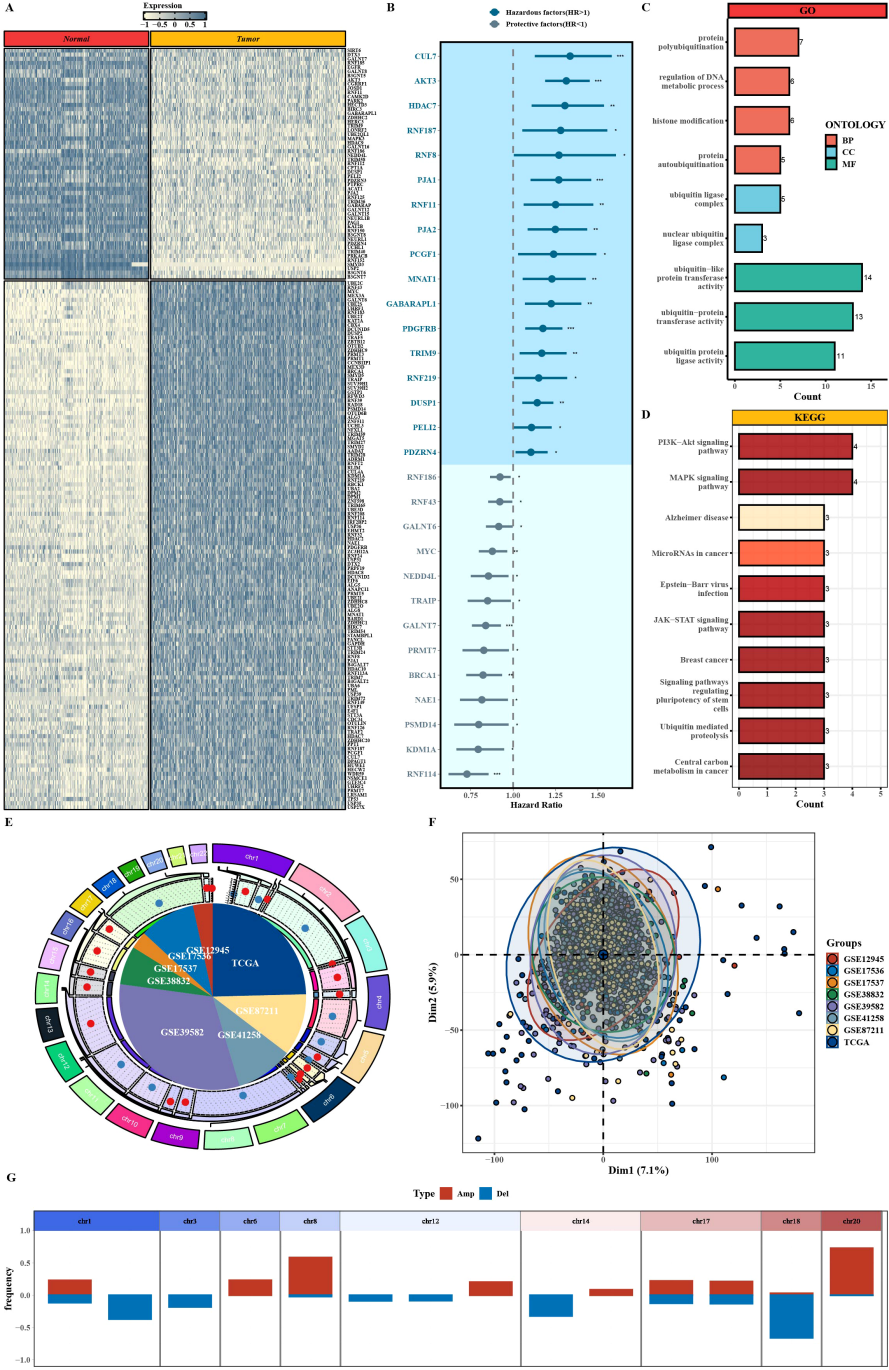
Screening and characterization of PTM-related differentially expressed genes. **(A)**The differential expression of PTM-related genes between tumor and normal tissues. **(B)** HR for 30 prognostic PTM-related genes and their relationship with survival in CRC patients. **(C, D)** Functional enrichment of prognostic PTM-related genes in GO biological processes and KEGG pathways. **(E)** Circos plot of PTM-related genes. **(F)** The principal component analysis of different datasets after batch effect correction. **(G)** The CNV frequency of prognostic PTM-related genes on chromosome arms, highlighting the prevalence of amplifications and deletions.

First, we integrated TCGA and GTEx colon datasets and performed differential expression analysis between tumor and adjacent normal tissues within a predefined list of PTM-related genes (thresholds: |log2FC| >0.6, P < 0.05), thereby identifying PTM-related DEGs. The heatmap showed that the selected PTM-related DEGs robustly separated tumors from normal samples, indicating stable expression heterogeneity in COAD (**Figure 1A**). Through univariate Cox regression analysis, 30 PTM-related genes significantly associated with the prognosis of CRC patients were identified from the PTM-related DEGs (**Figure 1B**). The forest plot indicates that genes such as CUL7, AKT3, and PDGFRB etc. act as hazardous factors (HR > 1). Functional enrichment analysis (**Figures 1C, D**) indicated that these prognosis-related genes were significantly enriched in core PTM-related processes, including protein polyubiquitination and histone modification in Gene Ontology (GO) biological processes. Kyoto Encyclopedia of Genes and Genomes (KEGG) pathway enrichment analysis showed that these genes were mainly involved in classic cancer-related signaling pathways, PI3K-AKT and mitogen-activated protein kinase (MAPK) signaling pathways. To verify the robustness of the model across populations and batches, batch correction was performed on TCGA and GTEx datasets. Principal component analysis (PCA) before and after correction confirmed that batch effects were effectively reduced, supporting the reliability of subsequent cross-dataset analyses (**Figure 1F**; **Supplementary Figure 1A**). Genomic variation analysis showed (**Figures 1E, G**) chromosome-level copy number variation (CNV) frequencies of PTM-related genes. Amplifications are more prevalent than deletions on several arms (notably chr8 and chr20), whereas losses are comparatively enriched on others (e.g., chr1, chr14, chr18). A heatmap further showed expression stratification and coherent expression trends of these core genes across clinical subgroups defined by age, stage, T/N/M category, and survival status, consistent with the overall differential expression patterns and prognostic directions (**Supplementary Figure 1B**). Together, these CNVs of PTM-related genes and expression pattern correlated with clinical features provided preliminary support for the clinical applicability.

### 3.2 Construction and integrated validation of a multi-algorithm PTMRS model and its clinical value analysis

**Figure 2:**
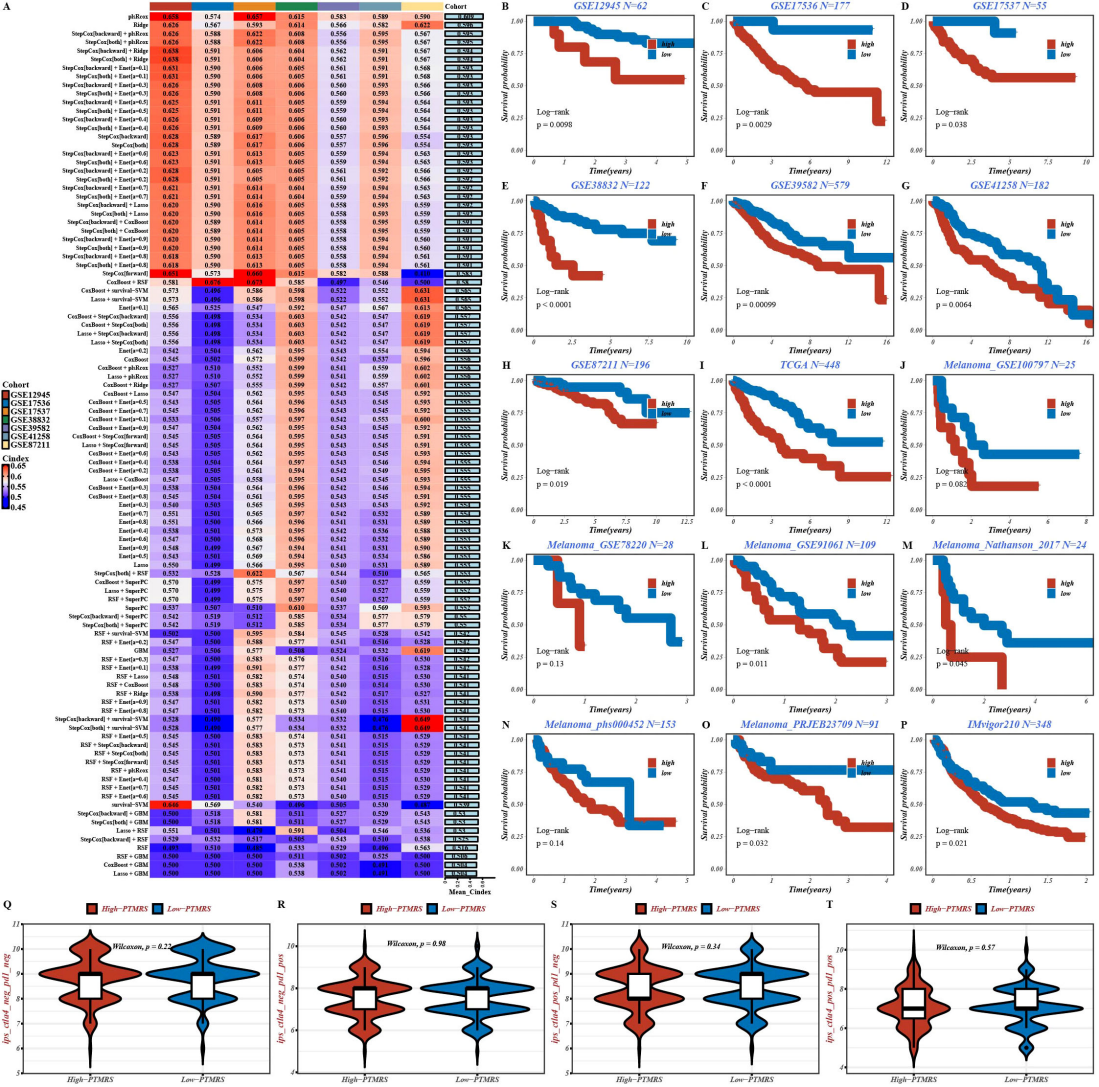
Construction and integrated validation of the PTMRS model. **(A)** C-index values for various algorithms used in the PTMRS model, comparing their predictive power. **(B-I)** Kaplan–Meier survival analysis evaluating the prognostic value of PTMRS in CRC patients(P<0.05). **(J-P)** Survival analysis on immunotherapy cohorts to validate the prognostic performance of PTMRS in immunotherapy (P<0.05). **(Q-T)** The differences in IPS between high-PTMRS and low-PTMRS groups.

Building on this candidate set, we benchmarked 101 modeling strategies and their combinations. Based on an integrated comparison of the concordance index (C-index) and time-dependent AUC, the plsRcox model was selected as the final framework for PTMRS construction (**Figure 2A**). PTMRS scores were then calculated for individuals in the TCGA-COAD training cohort, and patients were stratified into high-PTMRS and low-PTMRS groups using an optimal cutoff. Subsequently, the model was systematically evaluated across four dimensions: prognostic performance, pan-cancer applicability, molecular validation, and therapeutic implications.

In the TCGA-CRC training set (n = 448), Kaplan–Meier analysis showed that patients in the high-PTMRS group had significantly shorter overall survival (OS) than those in the low-PTMRS group (log-rank P < 0.0001; **Figure 2I**). To assess model stability, external validation was performed in seven independent GEO CRC cohorts, all of which showed consistent prognostic stratification, further supporting the robustness of PTMRS in CRC (**Figures 2B–H**). We further performed a cross-cancer assessment in immunotherapy-treated melanoma and urothelial carcinoma cohorts. Despite differences in disease spectra, PTMRS consistently separated survival across multiple datasets (**Figures 2J-P**), suggesting a degree of generalizability in capturing the “tumor stress–PTM–prognosis” axis. Notably, immunophenoscore (IPS) analysis did not reveal significant differences between the high-PTMRS and low-PTMRS groups across several immune phenotypes (**Figures 2Q–T**). However, the absence of marked IPS differences does not preclude immune involvement. It is plausible that the prognostic signal of PTMRS primarily reflects tumor-intrinsic proteostasis and stress-response pathways, while immune interactions may be more complex and context dependent, and thus not fully captured by IPS alone.

To further assess the biological relevance and therapeutic potential of PTMRS, we performed protein-level external validation. PTMRS was positively correlated with the abundance of several proteins (e.g., ELMO2, FERMT2, GPX4; **Supplementary Figures 2A–H**) involved in membrane–cytoskeleton coupling, redox regulation, and autophagy, suggesting that higher PTMRS scores are associated with an increased demand for proteostasis remodeling. By integrating gene-dependency datasets to assess functional essentiality, we found that PTMRS was broadly negatively correlated with the CERES scores of these genes (**Supplementary Figures 2I–P**), indicating that tumors with higher PTMRS scores show stronger dependence on these proteins. Based on drug–sensitivity association analyses, PTMRS was negatively correlated with the predicted AUCs of multiple agents (e.g., QS-11, azacitidine, myriocin, nelarabine, and two BRD-K compounds; **Supplementary Figure 2Q**). Consistently, comparisons between PTMRS strata showed that lower estimated AUCs in the high-PTMRS group for these drugs (**Supplementary Figure 2R**). Notably, for sepantronium bromide (compound 1941), PTMRS was negatively associated with drug response, and the high-PTMRS group exhibited lower estimated IC50 values (**Supplementary Figures 2S–T**). Taken together, these findings suggest that PTMRS may help prioritize mechanistic targets and support individualized treatment strategies.

### 3.3 Prognostic independence of PTMRS and its comprehensive evaluation across clinical factors, external validation cohorts, and genomic alterations

**Figure 3:**
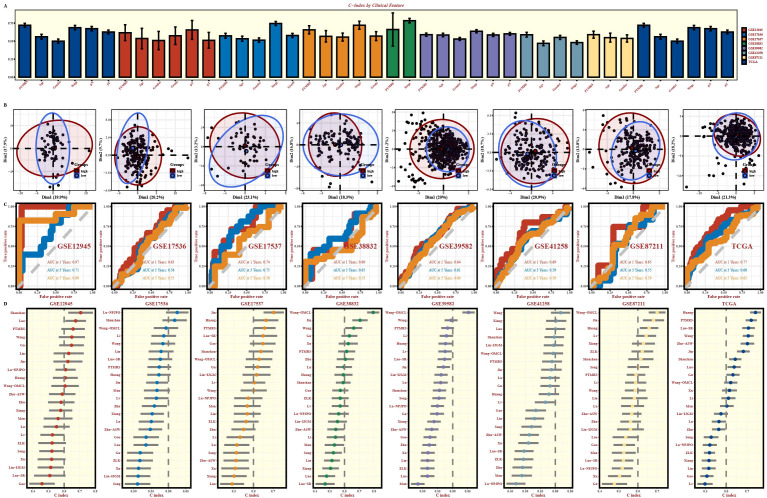
Comprehensive evaluation of the PTMRS model. **(A)** The C-index comparison of PTMRS with clinical variables (age, sex, pT, pN, and pathological stage). **(B, C)** PCA results and time-dependent ROC analysis for PTMRS risk groups in the COAD dataset. **(D)** The C-index comparison of PTMRS with 22 previously published prognostic models.

To assess clinical applicability, we compared PTMRS with common clinical variables (age, sex, stage, pT/pN, pathological grade) in terms of C-index. PTMRS ranked first or near the top in most cohorts and clearly outperformed any single clinical feature; for example, in GSE12945 and TCGA, its discriminative ability exceeded traditional variables such as age and stage, indicating that it provides independent prognostic information (**Figure 3A**).

PCA based on PTMRS stratification showed clear separation between high-PTMRS and low-PTMRS groups across datasets, suggesting that the signature captures stable and transferable molecular differences (**Figure 3B**). Time-dependent receiver operating characteristic (ROC) analyses further supported its predictive performance: the 1-, 3-, and 5-year areas under the curve (AUCs) were generally in the moderate-to-high range across cohorts—for instance, 0.97, 0.71, and 0.89 in GSE12945 and 0.77, 0.68, and 0.63 in TCGA—while most other datasets yielded AUCs between 0.55 and 0.75 (**Figure 3C**). In C-index comparisons with 22 previously published prognostic models, PTMRS ranked among the top performers or achieved the best performance in most external cohorts (**Figure 3D**). Taken together, PTMRS retains independent prognostic value beyond clinical variables and exhibits stable, generalizable survival discrimination across multicenter datasets.

GISTIC2.0 analysis (**Supplementary Figures 3A, B**) confirmed that the frequencies of chromosomal segment amplifications and deletions were significantly higher in the high-PTMRS group than in the low-PTMRS group. High-PTMRS tumors carried a heavier genomic alteration burden: frequent mutations in canonical CRC drivers (APC, TP53, KRAS, PIK3CA) co-occurred with recurrent segmental CNV gains and losses, and copy-number changes were biased toward gains, with elevated arm-level and focal amplifications compared with relatively weaker losses (**Supplementary Figures 3C, D**). Consistently, survival analysis demonstrated that patients in the high-PTMRS and high-TMB subgroup had the poorest overall survival(log-rank P<0.0001, **Supplementary Figure 3E**). These findings suggest that CNVs in PTM-related genes and TMB may act synergistically to influence CRC prognosis, underscoring a link between genomic instability and PTM-related regulation.

### 3.4 Association analysis of PTMRS with the tumor immune microenvironment and key signaling pathways

To elucidate how PTMRS may influence CRC outcomes, we examined three dimensions: the cellular composition of TIME, the expression of immune-related molecules, and the enrichment of key signaling pathways. These analyses outlined the associations between PTMRS, TIME remodeling, and pathway dysregulation.

**Figure 4:**
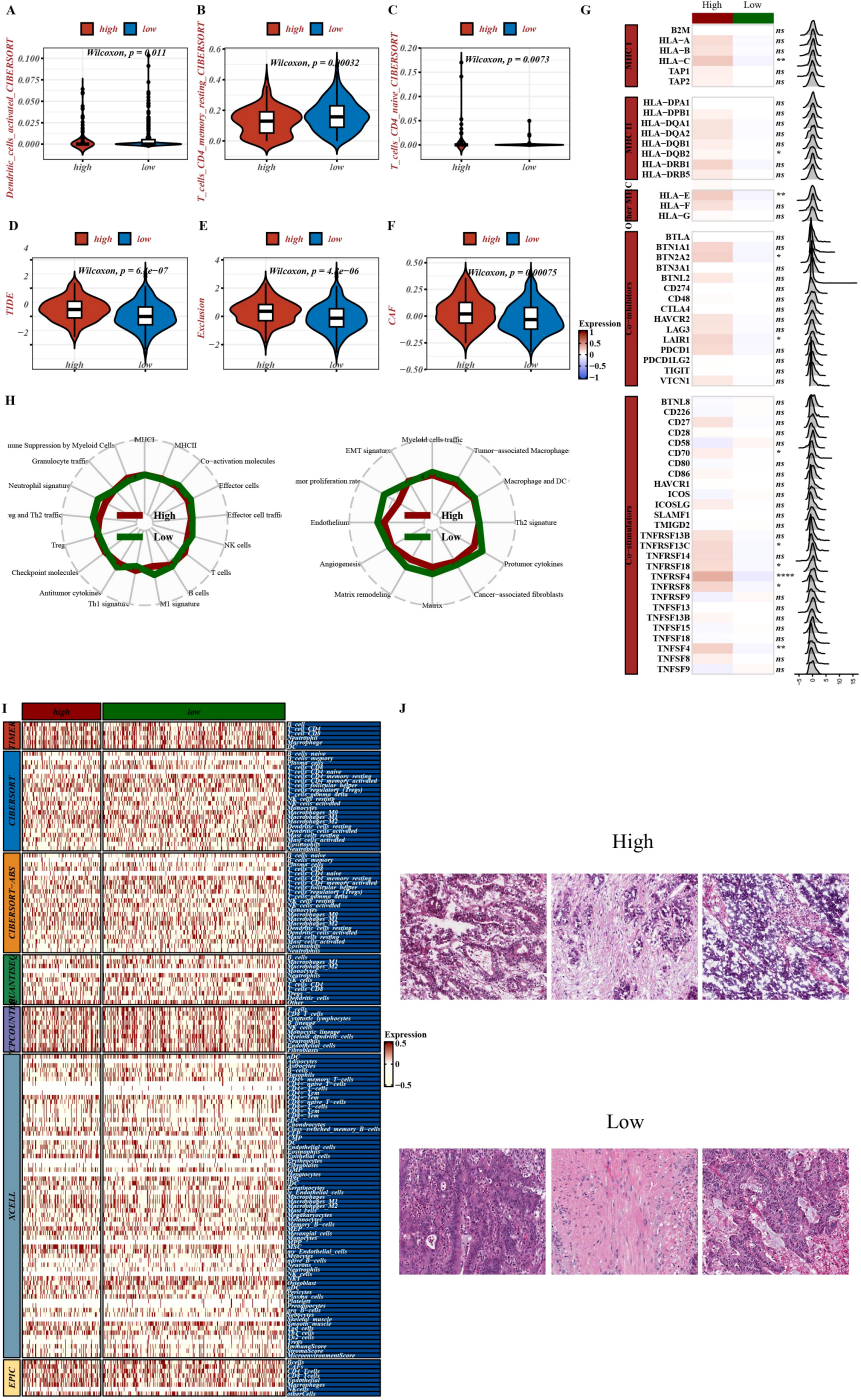
Immune-related analysis. **(A–F)** Estimated differences in tumor-infiltrating immune cell populations between high- and low-PTMRS groups as assessed by CIBERSORT and TIDE. **(G)** Correlations between PTMRS and the expression of immune-related genes. **(H)** Radar plot summarizing differences in immune-related pathways between high- and low-PTMRS groups. **(I)** Heatmap showing differential immune infiltration between high- and low-PTMRS groups across seven immune deconvolution tools. **(J)** Representative pathological images of colorectal cancers with high versus low PTMRS scores from the TCGA cohort.

From the perspective of inferred immune-cell composition and functional scoring, marked differences were observed between high-PTMRS and low-PTMRS groups across multiple immune indices: the overall Tumor Immune Dysfunction and Exclusion (TIDE) score, immune exclusion score, and cancer-associated fibroblast (CAF) abundance were all higher in the high-PTMRS group, indicating a more immunosuppressive, stroma-remodeled microenvironment (**Figures 4D–F**). In contrast, activated dendritic cells, resting memory CD^+^ T cells, and naive CD4^+^ T cells were enriched in the low-PTMRS group (**Figures 4A–C**), suggesting that a high PTMRS is associated with reduced effector immune infiltration and a shift toward suppressive or exhausted immune states.

At the molecular level (**Figure 4G**),PTMRS showed significant correlations with genes involved in key immune processes, including major histocompatibility complex class I and II (MHC I and MHC II; antigen presentation and immune recognition), co-stimulatory molecules, and immune checkpoint molecules. A radar plot (**Figure 4H**) further illustrated differences in immune-related pathways between PTMRS strata, highlighting strong associations between PTMRS and pathways related to T-cell recruitment, immune-cell trafficking, and immune activation. These results underscored the potential role of PTMRS in modulating immune responses within TIME. Broad immune-infiltration estimates aggregated from multiple algorithms (TIMER, CIBERSORT-ABS, QUANTISEQ, MCPcounter, xCell, EPIC; **Figure 4I**) showed concordant separation according to PTMRS status. Consistently, representative TCGA pathology slides (**Figure 4J**) suggested that low-PTMRS colorectal cancers tended to be better differentiated, with milder nuclear pleomorphism and more prominent inflammatory infiltration.

Functionally, PTMRS showed significant associations with ubiquitin-mediated proteolysis, fatty-acid metabolism, peroxisome pathways, and H3K27 methylation regulation, suggesting more active protein degradation, lipid–oxidative remodeling, and epigenetic reprogramming in the high-PTMRS group (**Supplementary Figures 4A–G**).In a broader pathway-correlation matrix, proteasome, DNA replication, and antigen presentation together with immune effector signatures (e.g., IFNG), formed a coherent network centered on PTMRS. Consistently, stepwise analyses within TIME showed systematic associations between PTMRS and multiple stages of immune-cell recruitment, activation, and effector functions, supporting its link to immune remodeling (**Supplementary Figure 4H**).

Taken together, high-PTMRS and low-PTMRS groups showed consistent differences in immune-cell composition, immunosuppressive status, gene-expression patterns, and immune infiltration estimated by multiple algorithms, with supportive evidence from histopathology and pathway analyses. These findings support PTMRS as a promising marker for immune stratification and potential therapeutic responsiveness.

### 3.5 Single-cell PTMRS scoring and cell communication features in the TIME of colorectal cancer

To delineate how the PTMRS is distributed across cell types within the CRC TME and how it influences intercellular communication, we analyzed two CRC scRNA-seq datasets. By integrating cell annotation, PTMRS scoring, and cell–cell communication analysis, we characterized the associations between PTMRS and cellular functions.

**Figure 5:**
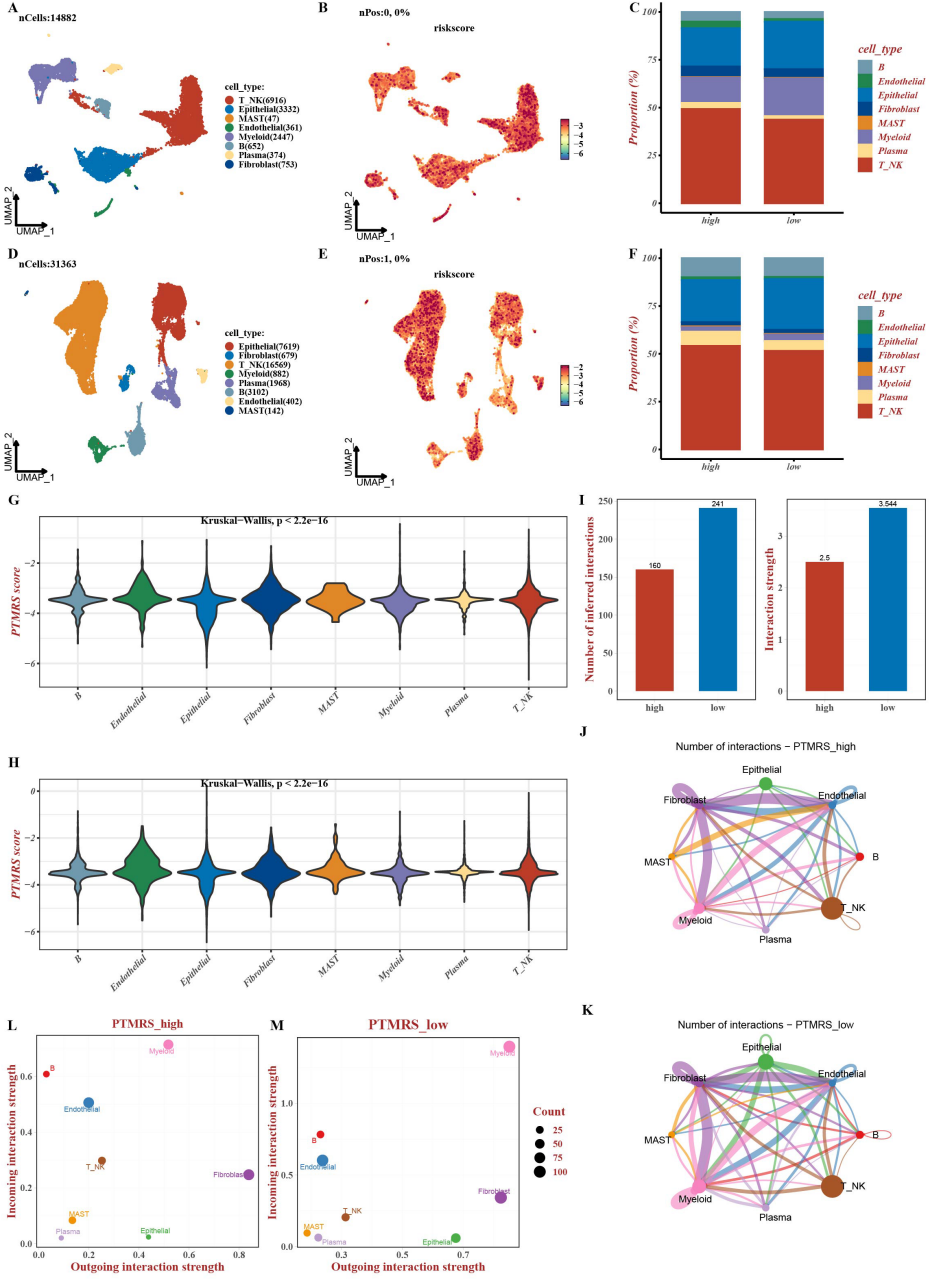
Single-cell analysis of PTMRS distribution and cell–cell communication. **(A–C)** GSE231559: **(A)** UMAP plot colored by annotated cell type; **(B)** UMAP plot colored by per-cell PTMRS; **(C)** bar plot showing cell-type composition in high-PTMRS versus low-PTMRS groups. **(D–F)** GSE200997: **(D)** UMAP plot colored by annotated cell type; **(E)** UMAP plot colored by per-cell PTMRS; **(F)** bar plot showing cell-type composition in high-PTMRS versus low-PTMRS groups. **(G–H)** Violin plots depicting PTMRS distributions across major cell types in both datasets (Kruskal–Wallis test, P < 2.2 × 10^-16^). **(I–M)** CellChat-based comparison of intercellular communication between high- and low-PTMRS groups, including overall network connectivity and cell type–specific incoming and outgoing signaling strengths.

We annotated epithelial cells (the primary source of tumor cells), stromal cells (including fibroblasts and endothelial cells), and immune populations (T/NK cells, B cells, plasma cells, myeloid cells, and mast cells). PTMRS scores were calculated for each cell and visualized using uniform manifold approximation and projection (UMAP). PTMRS was generally elevated across multiple lineages, with T/NK cells, B cells, and endothelial cells showing among the highest scores. After stratifying cells into high-PTMRS and low-PTMRS groups by the median score, the high-PTMRS group showed a lower proportion of epithelial cells and a higher proportion of T/NK and plasma cells (**Figures 5A–F**). Violin plots in both datasets further confirmed that endothelial and T/NK cells consistently exhibited higher PTMRS scores than other cell types (**Figures 5G–H**), indicating a cell type–biased distribution in which immune (T/NK, B) and vascular endothelial cells are major carriers of the PTMRS signal.

Using CellChat, we compared intercellular communication between t high-PTMRS and low-PTMRS groups and found that the high-PTMRS group exhibited fewer interactions than the low-PTMRS group (**Figure 5I**). Group-specific communication networks revealed reduced interactions between epithelial cells and other compartments in the high-PTMRS group (**Figures 5J–K**), with smaller node sizes for epithelial and plasma cells, consistent with weaker outgoing and incoming signaling (**Figures 5L–M**). A heatmap of efferent signal strength across eight cell types (including epithelial cells, fibroblasts, and T/NK cells) and key signaling pathways—such as macrophage migration inhibitory factor (MIF), vascular endothelial growth factor (VEGF), CXCL family members, and TGF-β—demonstrated distinct signaling profiles between PTMRS strata, with fibroblasts in the high-PTMRS group exhibiting particularly strong signaling outputs (**Supplementary Figure 5**).

### 3.6 PDGFRB as a central node in the PTMRS network: expression, survival, and immune correlates

Based on the multi-dimensional analysis described above, we found that PTMRS is strongly associated with the remodeling of TIME and abnormal intercellular interactions. Among the key genes comprising PTMRS, PDGFRB, which encodes PDGFRβ, emerged as a risk gene with a hazard ratio (HR) greater than 1 in the preliminary univariate Cox analysis. In this context, we defined central node genes as those that simultaneously (i) act as adverse prognostic factors in survival analyses, (ii) exhibit a positive correlation with the composite PTMRS, and (iii) functionally link to TME remodeling by upregulating immunosuppressive pathways. Among the 30 PTM-related prognostic genes, PDGFRB was the gene fulfilling all of these criteria across independent cohorts, which is why we refer to it as a central node within the PTMRS-related network. The receptor tyrosine kinase encoded by PDGFRB plays a pivotal role in tumor stroma activation and angiogenesis. Consequently, we focused our subsequent expression validation and functional analysis on PDGFRB to further elucidate its critical role within the PTMRS regulatory network.

**Figure 6:**
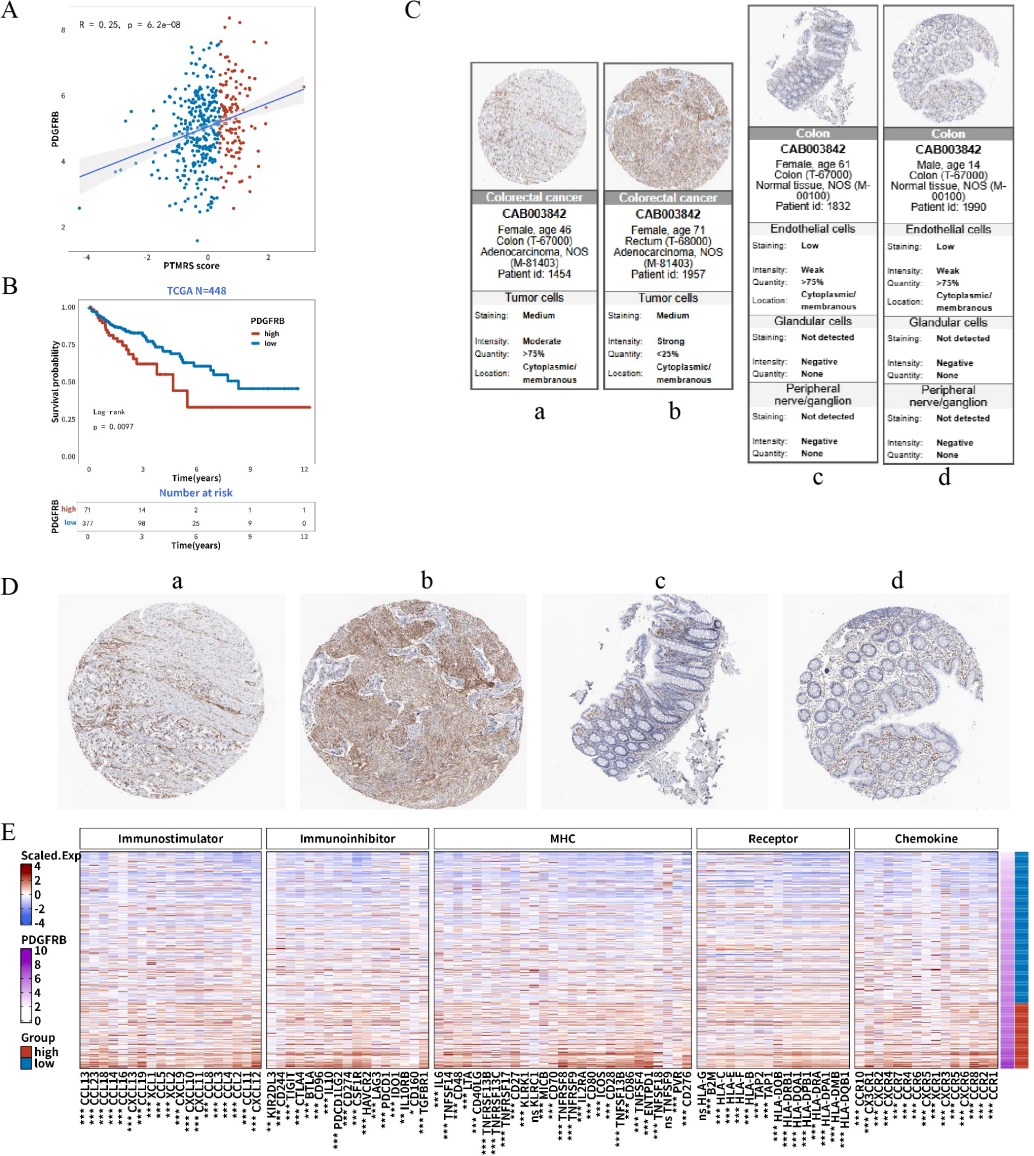
Analysis of PDGFRB expression and its correlation with immune-related pathways. **(A)** Correlation between PTMRS and PDGFRB expression levels in colorectal cancer samples, showing a significant positive correlation between PDGFRB expression and PTMRS (Pearson R = 0.25, P < 0.05). **(B)** Kaplan-Meier survival analysis of PDGFRB expression in the TCGA cohort, showing patients with low PDGFRB expression have significantly better overall survival compared to those with high expression (P < 0.05). **(C, D)** Immunohistochemical analysis of PDGFRβ protein expression in tumor and normal tissues from the HPA database, showing distinct differences in PDGFRB expression between tumor and normal tissues, with elevated expression in the tumor. **(E)** Heatmap of the correlation between PDGFRB and immune-related genes.

Correlation analysis indicated a significant positive association between PDGFRB expression and PTMRS (Pearson R = 0.25, P < 0.05; **Figure 6A**). In the TCGA cohort (N = 448), Kaplan–Meier analysis showed that patients with low PDGFRB expression had significantly better overall survival than those with high expression (P < 0.05; **Figure 6B**). Immunohistochemistry data from the HPA database reveal distinct differences between tumor and normal tissues (Figures C–D). A heatmap (**Figure 6E**) of correlations with immune-related genes further revealed upregulation of immunosuppressive pathways in the high-PDGFRB expression group: multiple inhibitory checkpoints and suppressive molecules were elevated, indicating a stronger immune brake. Taken together, *PDGFRB* appears to act as a central hub linking the “PTMRS–stromal activation–immunosuppression–poor prognosis” axis. It thus holds potential both as a prognostic biomarker and as a candidate therapeutic target, providing a clear rationale for further mechanistic studies and the development of PDGFRβ-directed interventions.

### 3.7 Impact of PDGFRβ perturbation within tumor-associated fibroblasts on CRC cell phenotypes

Based on the previous analysis of PDGFRB as a hub gene within the PTMRS network, its high expression is closely associated with poor prognosis and an immunosuppressive TME in CRC. It is plausible that PDGFRβ acts as a pivotal target mediating tumor–stroma crosstalk. To verify this hypothesis, we performed *in vitro* functional experiments to investigate how receptor activation versus targeted blockade reshape fibroblast signaling and, in turn, influence CRC cell proliferation and motility, thereby providing direct experimental evidence for this proposition.

**Figure 7:**
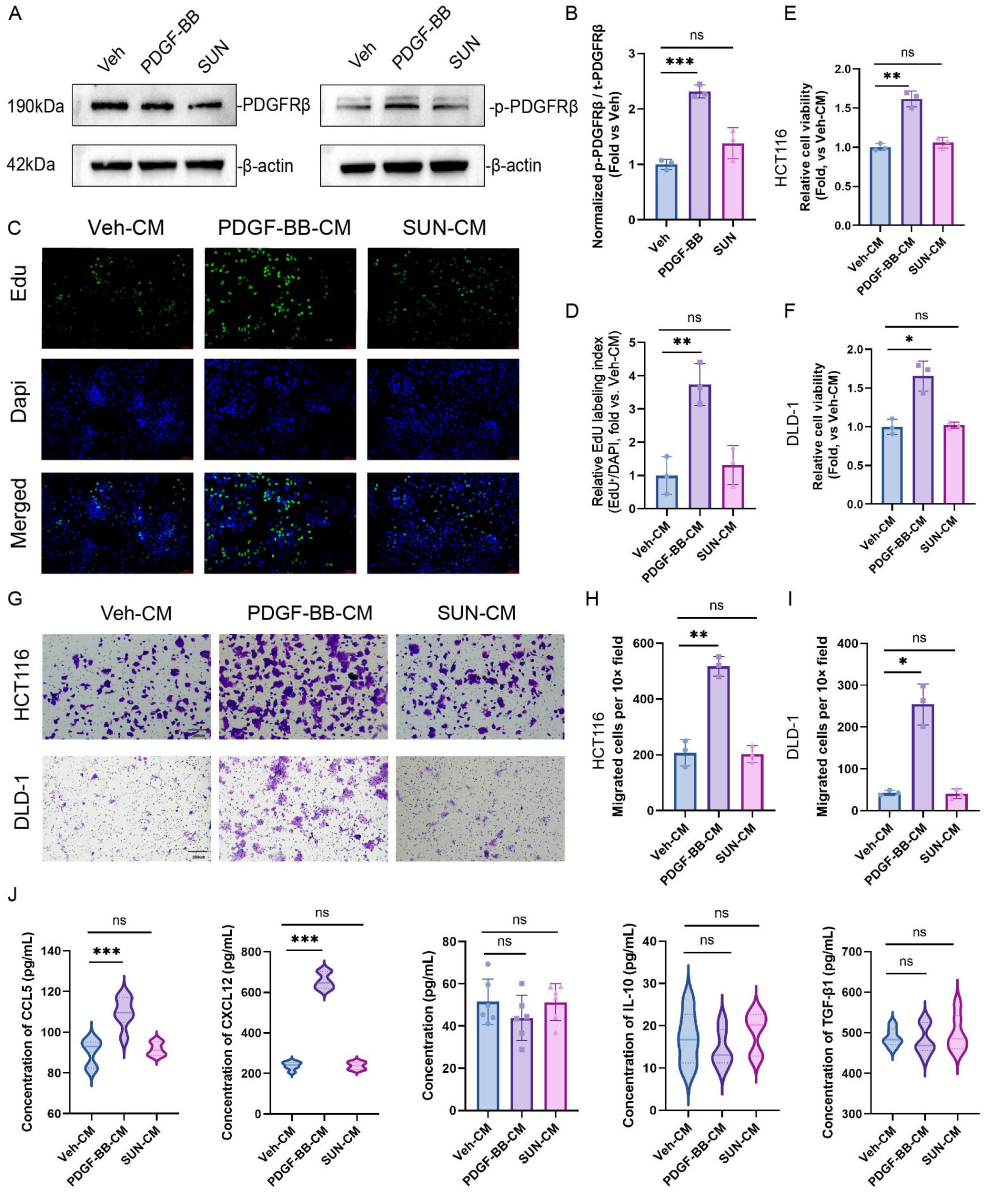
Impact of PDGFRb perturbation within tumor-associated fibroblasts on CRC cell phenotypes. **(A)** Western blots of t-PDGFRb and p-PDGFRb in CCD-18Co fibroblasts treated with vehicle (Veh), PDGF-BB, or PDGF-BB plus SUN. b-actin serves as a loading control. **(B)** Quantification of the p-PDGFRb/t-PDGFRb ratio relative to the Veh group. **(C)** Representative EdU staining of HCT116 cells cultured with CM from the three fibroblast conditions (Veh-CM, PDGF-BB-CM, SUN-CM). EdU (green), DAPI (blue), merged images as indicated. **(D)** Relative EdU labeling index in HCT116 cells. **(E, F)** CCK-8 assays showing relative cell viability of HCT116 **(E)** and DLD-1 **(F)** cells after 48 h exposure to Veh-CM, PDGF-BB-CM, or SUN-CM. **(G)** Representative Transwell images of migrated HCT116 and DLD-1 cells under the indicated CM conditions. **(H, I)** Quantification of migrated HCT116 **(H)** and DLD-1 **(I)** cells per 10× field. **(J)** Violin plots showing concentrations of CCL5, CXCL12, IL-6, IL-10, and TGF-b1 in fibroblast CM measured by ELISA. *P < 0.05, **P < 0.01, ***P < 0.001 vs Veh group; ns, no significant difference. Abbreviations: Veh, vehicle; SUN, sunitinib; CM, conditioned medium; PDGF-BB, the BB homodimer of platelet-derived growth factor.

To define the activation mechanism of PDGFRβ and the efficacy of its inhibitor, CCD-18Co cells were divided into three groups: vehicle control (Veh), PDGF-BB (ligand-mediated activation), and PDGF-BB plus sunitinib (SUN, a tyrosine kinase inhibitor of PDGFRβ).Western blotting (**Figure 7A**) showed a clear increase in phosphorylated PDGFRβ (p-PDGFRβ) after PDGF-BB stimulation, with total PDGFRβ (t-PDGFRβ) largely unchanged, indicating effective pathway activation in fibroblasts. Co-treatment with sunitinib markedly reduced p-PDGFRβ toward Veh levels. Quantitative analysis of band intensities confirmed this effect (**Figure 7B**): the p-PDGFRβ/t-PDGFRβ ratio was significantly higher in the PDGF-BB group than in the Veh group (P < 0.001) but decreased after sunitinib treatment and was no longer different from Veh (ns), indicating efficient and reversible inhibition of PDGFRβ activation.

Given the paracrine role of fibroblasts in shaping the TME, CM from the three fibroblast conditions were applied to CRC cells. In HCT116 cells, EdU staining showed that PDGF-BB CM significantly increased the EdU labeling index compared with Veh CM (P < 0.01; **Figures 7C–D**), whereas SUN CM reduced EdU incorporation back to a level comparable to Veh CM (P > 0.05, ns), indicating that sunitinib attenuates the PDGFRβ-driven proliferative signal. CCK-8 assays yielded consistent results in both HCT116 and DLD-1 cell lines: PDGF-BB CM significantly enhanced cell viability relative to Veh CM (P < 0.05; **Figures 7E–F**), while SUN CM brought viability back toward baseline, with no significant difference from Veh CM. Transwell assays were then used to assess migration. In HCT116 and DLD-1 cells, PDGF-BB-CM led to a marked increase in migrated cells compared with Veh-CM (P < 0.05; **Figures 7G–I**), whereas SUN-CM reduced migration toward Veh-CM levels.

To identify soluble mediators underlying these phenotypes, we measured cytokines and chemokines in fibroblast CM. Violin plots (**Figure 7J**) showed that CCL5 and CXCL12 were significantly elevated in PDGF-BB CM compared with Veh CM (both P < 0.001), whereas their levels in SUN CM were not different from Veh CM (P > 0.05, ns). In contrast, IL-6, IL-10, and TGF-β1 did not differ among the three groups (P > 0.05, ns). These data suggest that PDGFRβ activation preferentially upregulates specific chemokines, particularly CCL5 and CXCL12, which may contribute to the enhanced proliferation and migration of CRC cells.

In summary, signaling and functional assays in CRC cell lines showed that PDGF-BB–mediated PDGFRβ activation remodels the secretome of tumor-associated fibroblasts—especially by increasing the release of CCL5 and CXCL12—thereby promoting CRC cell proliferation and migration. Sunitinib blocks PDGFRβ activation and diminishes these tumor-promoting effects. These results further support PDGFRB as a stromal target aligned with the high-PTMRS phenotype and of potential therapeutic relevance in stroma-rich, high-PTMRS CRC.

## 4 Discussion

This study centers on a PTMRS model that integrates three pillars: molecular modification signals, TIME, and clinical outcomes. Higher PTMRS scores are associated with poorer overall survival, and high-PTMRS tumors typically show enhanced stromal activity (e.g., increased CAF signals), reduced immune infiltration, and a greater burden of chromosomal copy-number gains—features consistent with an immune-cold phenotype.

Although TIDE and several immune-infiltration algorithms indicate an immune-excluded, CAF-enriched microenvironment in the high-PTMRS group, Immunophenoscore (IPS) does not differ markedly between PTMRS strata, suggesting that IPS and PTMRS capture distinct biological dimensions. IPS is a widely used composite metric that estimates the likelihood of response to immune checkpoint inhibitors (ICIs) by integrating the expression of checkpoint molecules (e.g., programmed death-ligand 1, PD-L1 and cytotoxic T-lymphocyte–associated protein 4,CTLA-4) and effector factors (e.g., interferon-γ, IFN-γ and tumor necrosis factor-α, TNF-α) (16). However, IPS does not explicitly capture stroma-mediated immune barriers or PTM-driven interactions among epithelial, stromal, and immune cells—key features reflected by the PTMRS. As a result, tumors with high PTMRS may have IPS values similar to those with low PTMRS. However, they can still remain immunologically excluded, because PTM-driven fibroblast activation and extracellular matrix remodeling create an immune-excluding microenvironment that is not captured by IPS.

To better characterize the heterogeneity of CRC, Justin Guinney et al. proposed the Consensus Molecular Subtypes (CMS) classification, a robust and biologically interpretable framework. Among these, CMS4 is marked by pronounced stromal activation—robust TGF-β signaling, dense stromal infiltration, and increased angiogenesis—and typically displays immune exclusion with poor therapeutic responsiveness (17). In our cohort, the high-PTMRS group, enriched for stromal signals and biased toward broad copy-number amplifications, closely aligns with the CMS4 phenotype. This concordance provides external support for PTMRS and improves its biological and clinical interpretability. It also suggests that PTMRS may be useful for patient stratification and for guiding subtype-oriented interventions. PTMRS does not simply reproduce existing CMS or immune subtype classifications. By explicitly prioritizing PTM-related genes and pathways, it links stromal activation with immune dysregulation and should be considered a complement to CMS and immune classifications, with potential to be integrated with these systems for more refined risk stratification rather than to replace them. Using GISTIC2.0 (18), we identified chromosomal regions preferentially amplified in high-PTMRS tumors, pointing to potential driver loci and therapeutic targets. In addition, mapping high-PTMRS expression patterns onto CellChat ligand–receptor networks revealed weakened communication between fibroblasts and immune cells, consistent with the immune-cold CRC TIME described in recent single-cell and spatial omics studies (19, 20).

Within the stromal biomarker landscape, PDGFRβ is tightly linked to stromal activation, aberrant angiogenesis, and restricted immune infiltration. Numerous studies indicate that a higher abundance of PDGFRβ-positive cancer-associated fibroblasts (CAFs) is associated with worse outcomes (21–23). Although the classification and functions of CAF subsets remain debated across tumor types, multiple investigations in gastrointestinal malignancies consistently report that excessive stromal activation correlates with limited responses to immunotherapy (24–26). These findings suggest that *PDGFRB* may serve as a key bridge between the high-PTMRS state and an immunosuppressive TME. In patients with elevated PTMRS and PDGFRB enrichment, combining multitarget PDGFR inhibitors or indirect CAF-modulating strategies with immunotherapy may improve efficacy.

Although microsatellite instability–high (MSI-H) and deficient mismatch repair (dMMR) status are established biomarkers for identifying CRC patients likely to benefit from immune checkpoint inhibitor therapy, their overall prevalence is relatively low (27–29), and most CRCs are microsatellite stable (MSS) with poor responses. PTMRS may provide a complementary readout to MSI and tumor TMB: rather than quantifying genomic instability itself, it reflects the functional state of immune suppression and PTM-related programs. Prior studies have shown that PTMs of PD-L1—such as glycosylation and deubiquitination—can alter its stability and promote immune evasion (30, 31). Moreover, epidermal growth factor (EGF)-glycogen synthase kinase 3β (GSK3β)-driven PD-L1 glycosylation further augments immunosuppression (32). This mechanistic context may help explain why high-PTMRS tumors preferentially modulate checkpoint signaling and antigen-presentation pathways, thereby facilitating immune escape.

We acknowledge several limitations. First, inter-cohort heterogeneity may affect our findings: differences in platforms and workflows can introduce bias that may persist despite normalization and batch-effect correction. Second, although we have conceptually positioned PTMRS for comparison with established CRC classifiers such as the CMS, this preliminary study has not included comprehensive quantitative benchmarking—for example, formal correlation analyses with CMS1–4 labels or head-to-head comparisons with published proteostasis scores. Systematic integration and comparison of PTMRS with these molecular frameworks will be an important direction for future work and may clarify whether PTMRS can improve risk prediction and treatment decision-making for specific CMS or immune subgroups. Third, our experimental validation is confined to exploratory *in vitro* assays. While PDGFRβ-targeted perturbation in fibroblasts altered CRC cell proliferation and migration, we did not delineate downstream signaling pathways in tumor cells. Moreover, *in vivo* corroboration remains lacking: the CM system merely simulates paracrine crosstalk, and we have not assessed PDGFRβ inhibitors in animal models to evaluate their effects on tumor growth, metastasis, or tumor microenvironment remodeling. Future studies integrating *in vivo* validation will therefore be critical to confirming the efficacy and safety of PDGFRβ-targeted strategies in high-PTMRS CRC, and thereby supporting their clinical translation.

In future investigations, PTM-focused omics (PTMomics)—including phosphorylation, acetylation, ubiquitination, and glycosylation—can be applied to high-PTMRS and low-PTMRS tumors to verify transcriptomic findings and identify druggable enzymes (e.g., deubiquitinases [DUBs], lysine acetyltransferases [KATs]) as targets within the tumor stroma and the TME.

Furthermore, *in vivo* studies are needed to map the PDGFRβ-centered PTM network in stromal cells and TME, to clarify its contribution to CRC progression, and to evaluate whether inhibiting these pathways—alone or in combination with immunotherapy—can improve tumor control. For clinical translation, PTMRS could be integrated with established biomarkers such as MSI, TMB, and Immunoscore in prospective cohorts to determine its added value in predicting responses to immune checkpoint blockade (ICB) or multi-agent regimens. In high-PTMRS tumors, profiling PD-L1/PD-L2 PTM and targeting their regulatory enzymes may clarify whether modulating these PTMs can restore antigen presentation and T-cell function.

## 5 Conclusion

PTMRS incorporates the dimension of PTMs into CRC risk stratification and serves as a robust model for prognostic assessment. By integrating chromosomal instability, single-cell signaling features, and TIME, this model has a relatively clear mechanistic basis and shows potential clinical applicability. CRC patients with high PTMRS and marked PDGFRβ enrichment have particularly poor outcomes. Our functional experiments indicate that targeting PDGFRβ—thereby modulating stromal components of the TME, immune mediators, and tumor cell signaling—can substantially affect CRC cell growth and progression. In the future, combining immunotherapy with agents that target key PTM-related pathways may further enhance treatment efficacy. These hypotheses warrant validation in *in vivo* functional studies and in prospective clinical cohorts with detailed treatment-outcome data, which will be essential to strengthen the translational value of PTMRS and ultimately improve prognosis for patients with CRC.

## Data Availability

The datasets presented in this study can be found in online repositories. The names of the repository/repositories and accession number(s) can be found in the article/**Supplementary Material**.
