# Supplementary material for: From multi-omics to functional validation: the PTMRS stratifies TME and positions PDGFRB in CRC biology
Source: Front Immunol. 2026 Jan 12;16:1728291. doi: 10.3389/fimmu.2025.1728291 (PMC12832729; doi:10.3389/fimmu.2025.1728291)
Supplement: Supplementary file 1 [file DataSheet1.zip › Supplementary data/Legends of supplementary figures.docx]

**Supplementary figure 1**

(A) The principal component analysis results before and after batch effect correction. (B) Expression patterns of model genes across different clinical subgroups.

**Supplementary figure 2**

(A-H) The correlation between PTMRS and multiple proteins. (I-P)Correlation between PTMRS and gene dependency data (CERES), indicating stronger protein dependencies in high-risk tumors. (Q-T) Correlation between PTMRS and drugs from the GDSCv2 and CTRP databasese, valuating its potential for guiding individualized treatment.

**Supplementary Figure 3**

(A-B) CNV results from GISTIC2.0 software for high- and low-PTMRS groups in TCGA. (C) Mutation data from TCGA using maftools and CNV results from GISTIC2.0, visualized with ComplexHeatmap. (D) CNV gain and loss from both broad and focal perspectives between high- and low-PTMRS groups using GISTIC2.0. (E) TMB values based on TCGA mutation data and PTMRS groups to perform survival analysis across four prognostic subgroups(P<0.0001).

**Supplementary Figure 4**

(A-C) Single-cell PTMRS analysis results from the GSE231559 dataset, depicting the distribution of PTMRS scores across different cell types within the CRC TME, with T/NK cells, B cells, and endothelial cells exhibiting the highest PTMRS values in the high-PTMRS group. (D-F) PTMRS analysis results from the GSE200997 dataset, which corroborates the findings in GSE231559, demonstrating similar patterns of high PTMRS in immune-related and endothelial cells. (G-H) Violin plots of PTMRS scores from both the GSE231559 and GSE200997 datasets, showing the cell type-specific distribution of PTMR. (I-M) Intercellular communication differences between the high- and low-PTMRS groups as assessed by CellChat analysis.

**Supplementary Figure 5**

Heatmap of efferent signal intensities across different cell types in high- and low-PTMRS groups
