# Supplementary figures and images for: From multi-omics to functional validation: the PTMRS stratifies TME and positions PDGFRB in CRC biology
Source: Front Immunol. 2026 Jan 12;16:1728291. doi: 10.3389/fimmu.2025.1728291 (PMC12832729; doi:10.3389/fimmu.2025.1728291)

**A**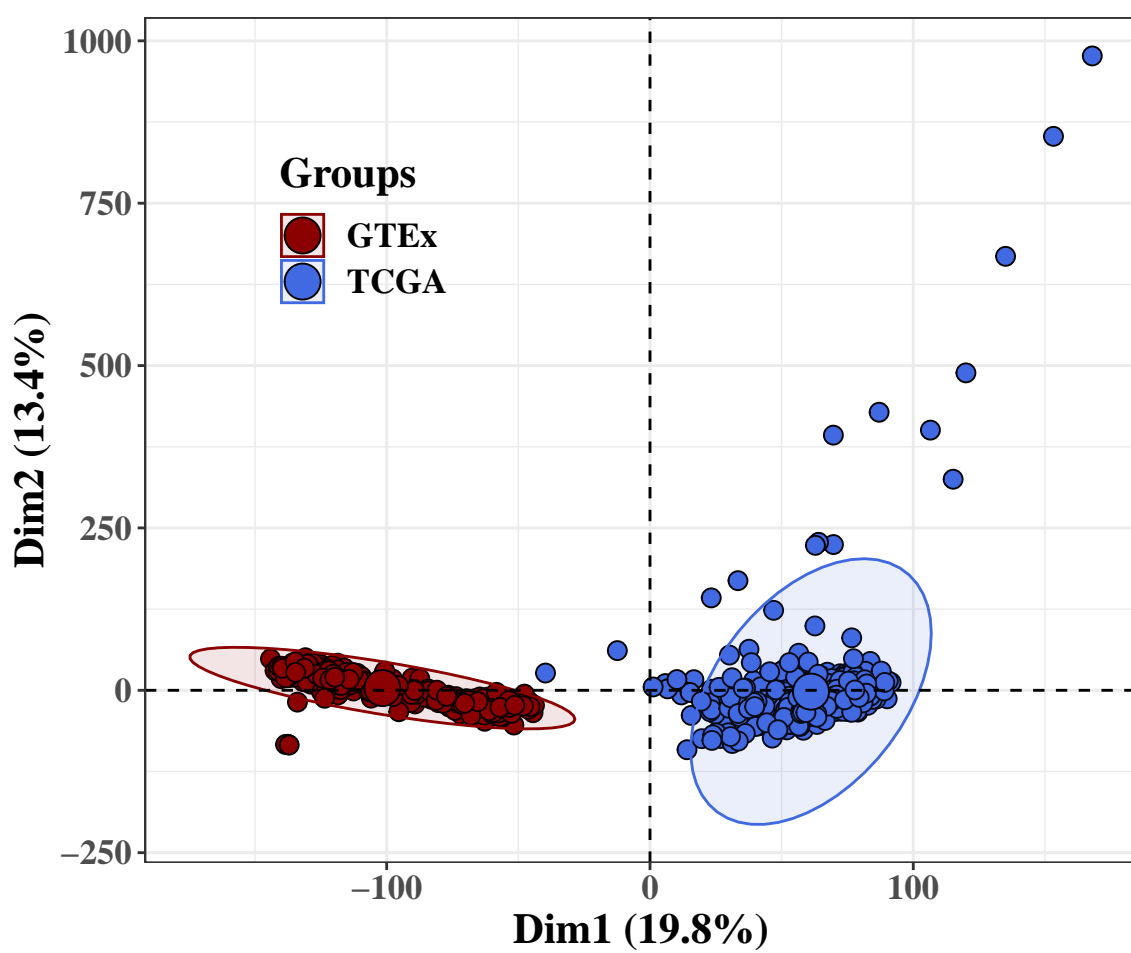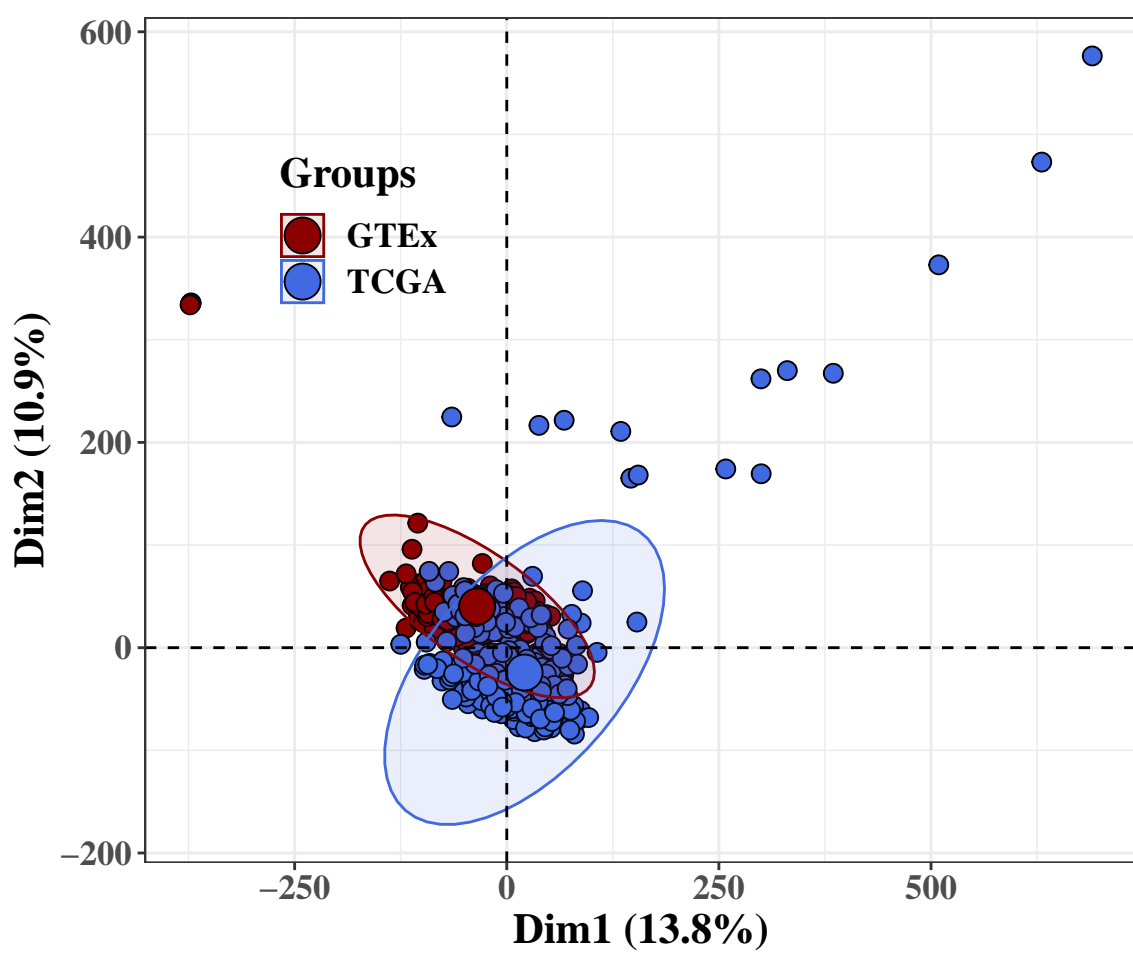**B**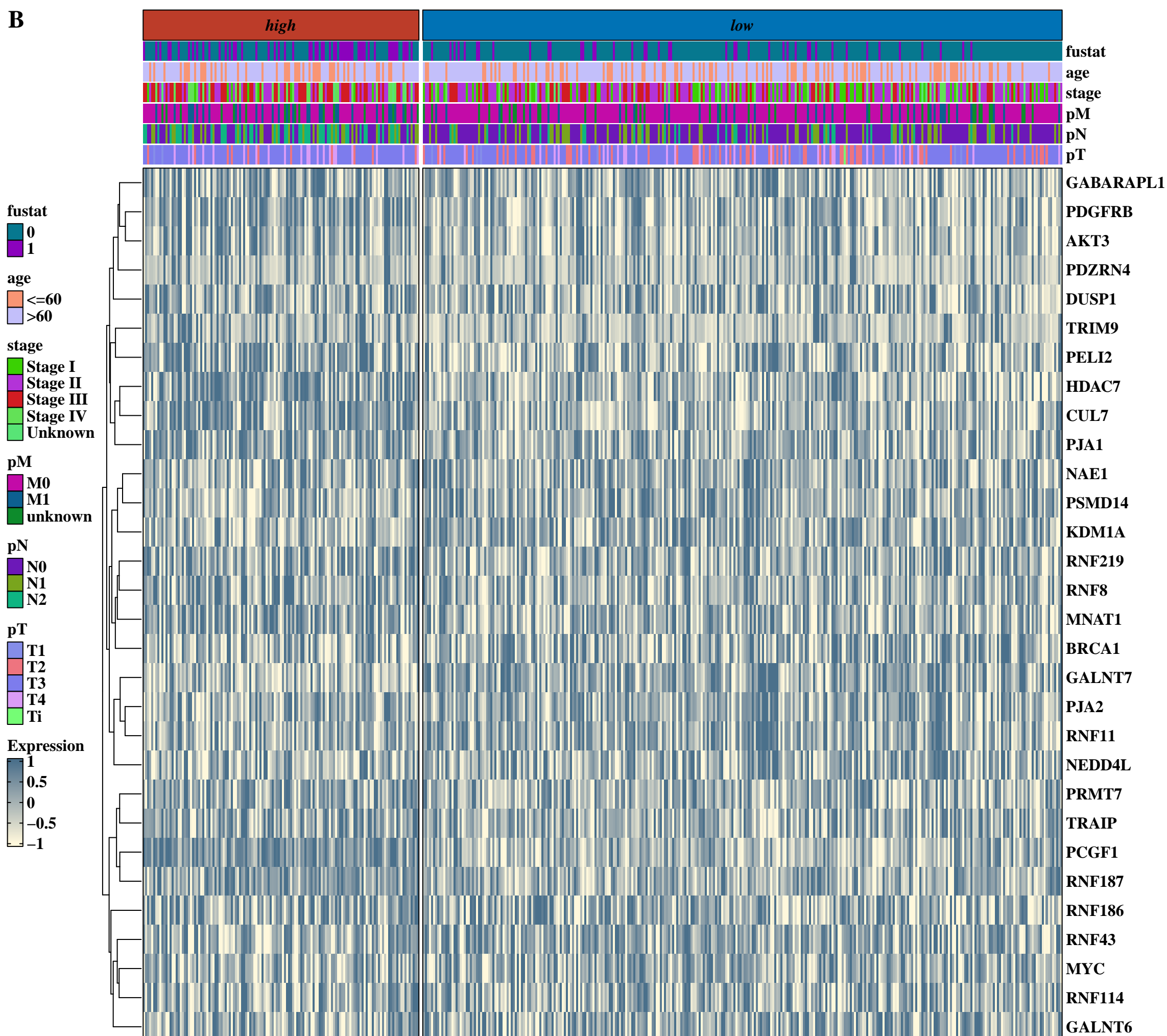

Supplement: Supplementary file 1 [file DataSheet1.zip › Supplementary data/Supfig1.pdf]

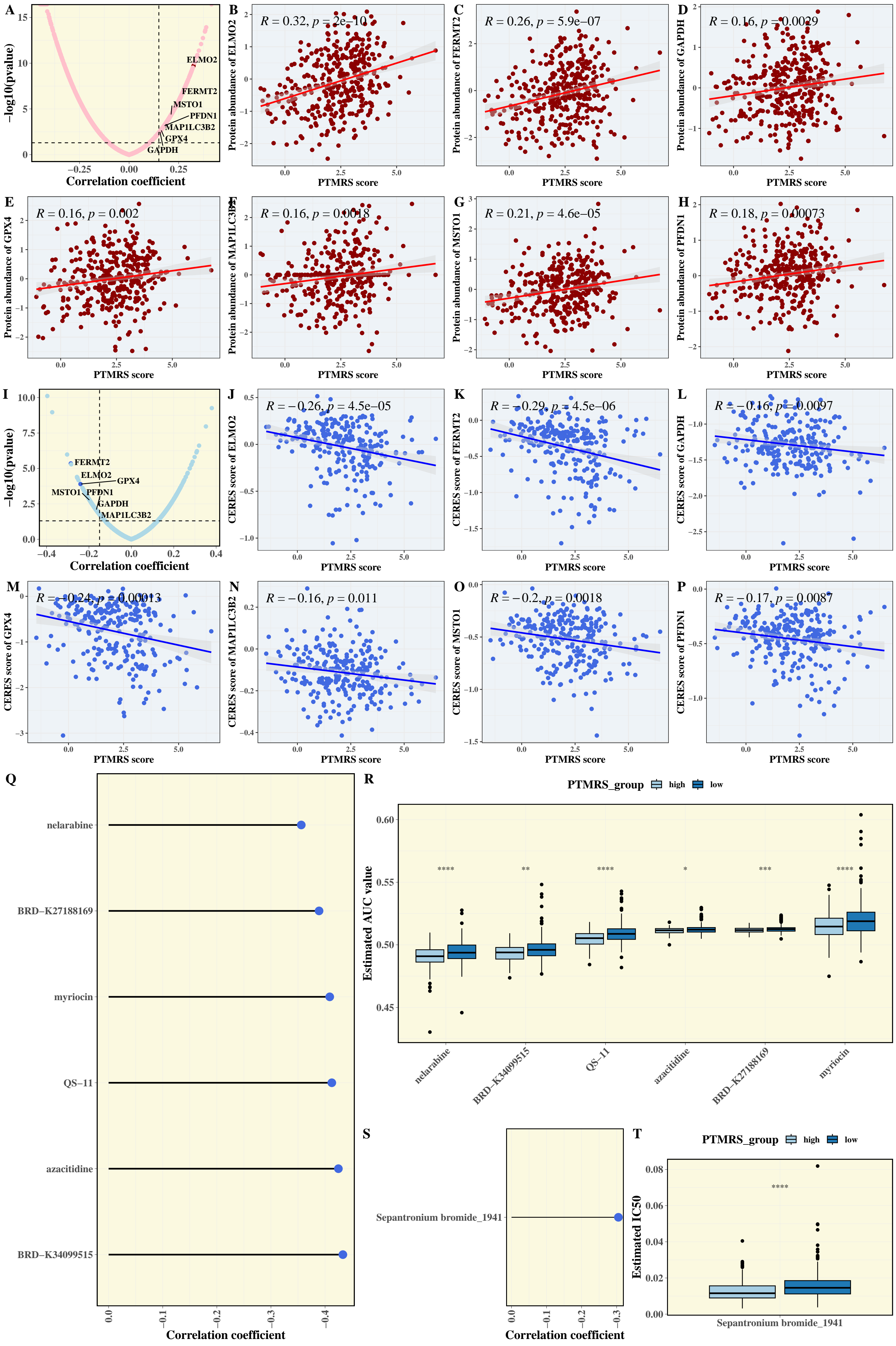

Supplement: Supplementary file 1 [file DataSheet1.zip › Supplementary data/Supfig2.pdf]

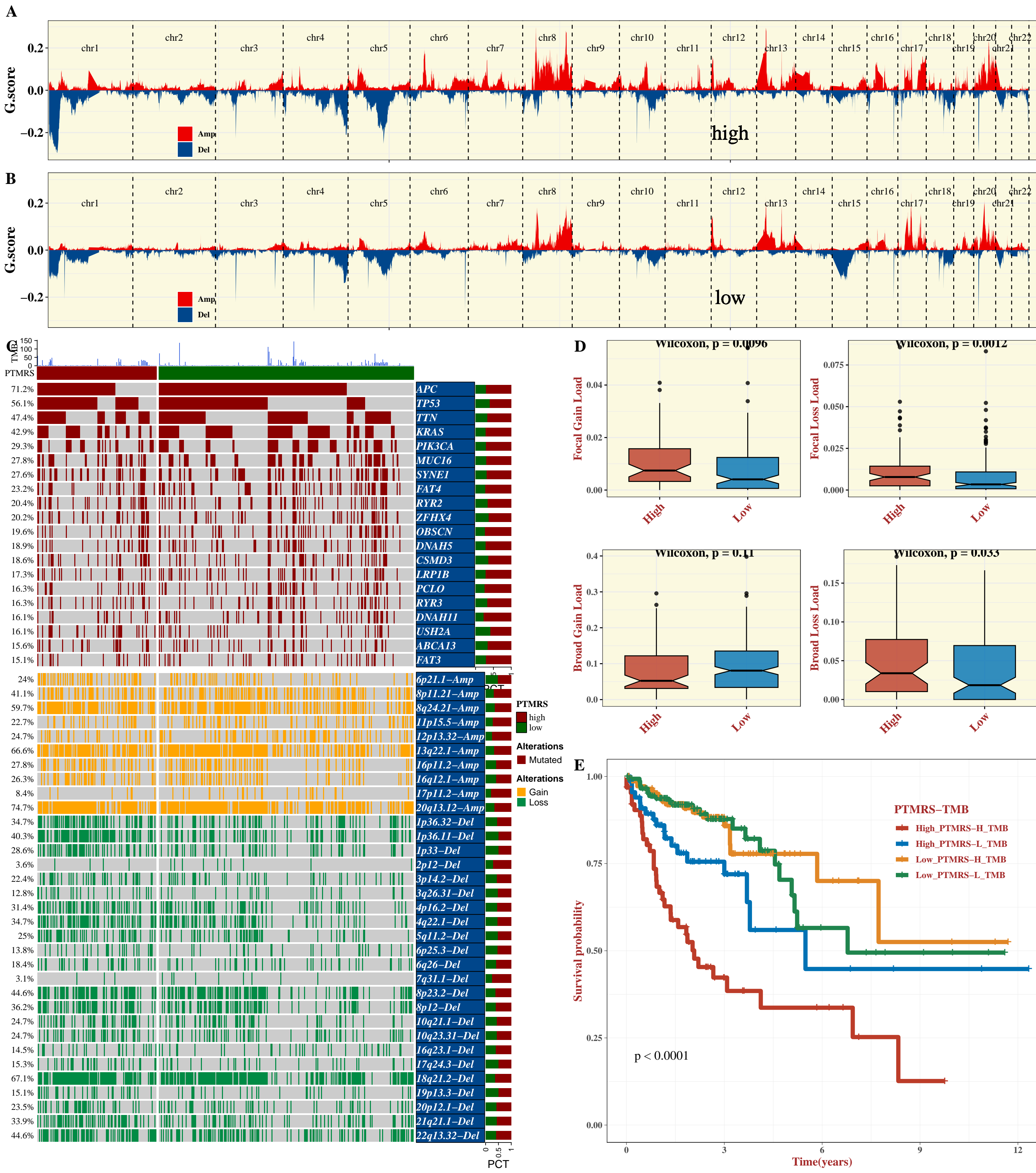

Supplement: Supplementary file 1 [file DataSheet1.zip › Supplementary data/Supfig3.pdf]
