# Supplementary material for: From multi-omics to functional validation: the PTMRS stratifies TME and positions PDGFRB in CRC biology
Source: Front Immunol. 2026 Jan 12;16:1728291. doi: 10.3389/fimmu.2025.1728291 (PMC12832729; doi:10.3389/fimmu.2025.1728291)
Supplement: Supplementary file 1 [file Supfig4.pdf › Supplementary data/Supfig4.pdf]

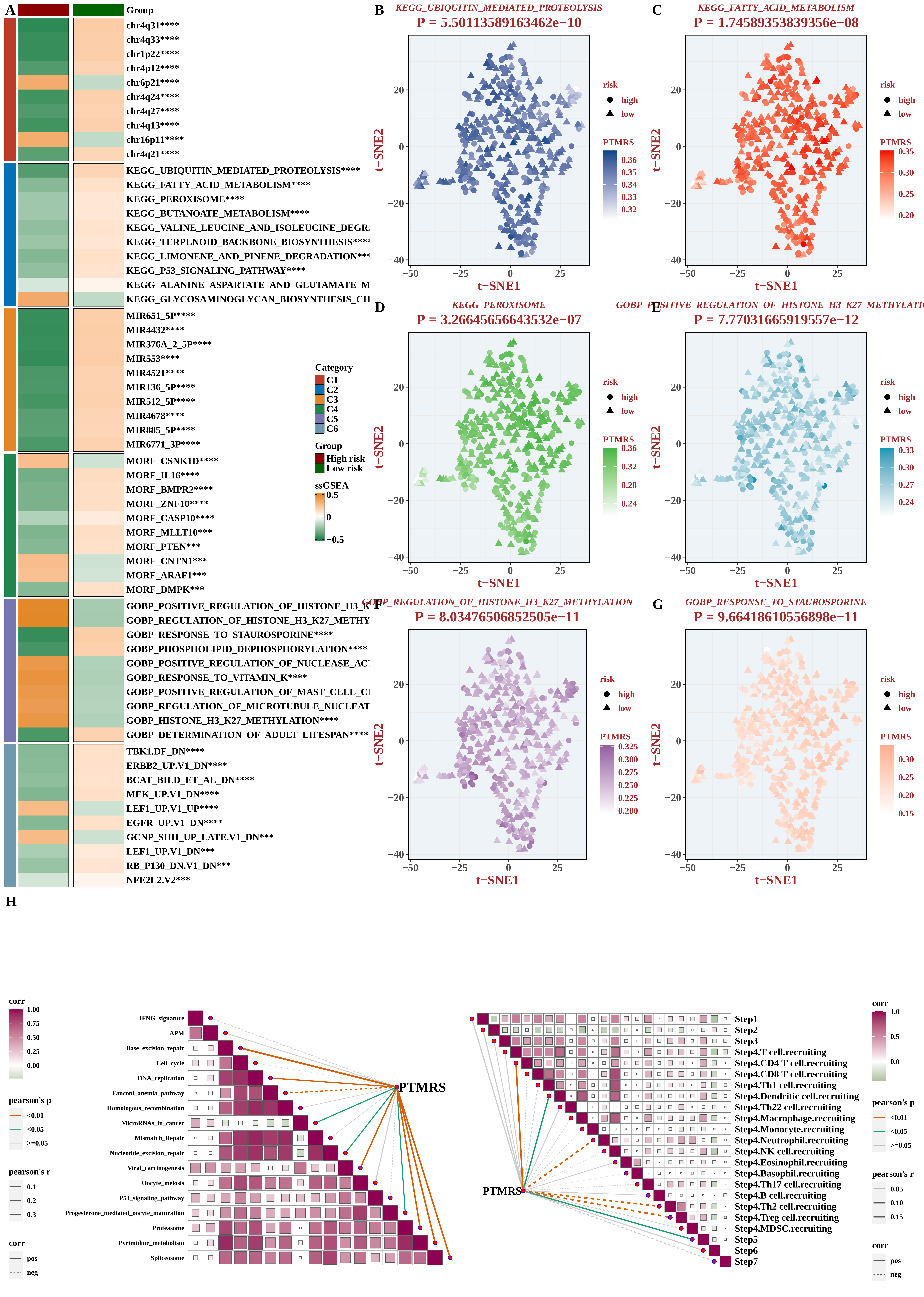

A
Group
chr4q31****
chr4q33****
chr1p22****
chr4p12****
chr6p21****
chr4q24****
chr4q27****
chr4q13****
chr16p11****
chr4q21****
KEGG_UBIQUITIN_MEDIATED_PROTEOLYSIS****
KEGG_FATTY_ACID_METABOLISM****
KEGG_PEROXISOME****
KEGG_BUTANOATE_METABOLISM****
KEGG_VALINE_LEUCINE_AND_ISOLEUCINE_DEGR
KEGG_TERPENOID_BACKBONE_BIOSYNTHESIS****
KEGG_LIMONENE_AND_PINENE_DEGRADATION***
KEGG_P53_SIGNALING_PATHWAY****
KEGG_ALANINE_ASPARTATE_AND_GLUTAMATE_M
KEGG_GLYCOSAMINOGLYCAN_BIOSYNTHESIS_CH
MIR651_5P****
MIR4432****
MIR376A_2_5P****
MIR553****
MIR4521****
MIR136_5P****
MIR512_5P****
MIR4678****
MIR885_5P****
MIR6771_3P****
MORF_CSNK1D****
MORF_IL16****
MORF_BMPR2****
MORF_ZNF10****
MORF_CASP10****
MORF_MLLT10****
MORF_PTEN***
MORF_CNTN1***
MORF_ARAF1***
MORF_DMPK***
GOBP_POSITIVE_REGULATION_OF_HISTONE_H3_K
GOBP_REGULATION_OF_HISTONE_H3_K27_METHY
GOBP_RESPONSE_TO_STAUROSPORINE****
GOBP_PHOSPHOLIPID_DEPHOSPHORYLATION****
GOBP_POSITIVE_REGULATION_OF_NUCLEASE_AC
GOBP_RESPONSE_TO_VITAMIN_K****
GOBP_POSITIVE_REGULATION_OF_MAST_CELL_C
GOBP_REGULATION_OF_MICROTUBULE_NUCLEAT
GOBP_HISTONE_H3_K27_METHYLATION****
GOBP_DETERMINATION_OF_ADULT_LIFESPAN****
TBK1.DF_DN****
ERBB2_UP.V1_DN****
BCAT_BILD_ET_AL_DN****
MEK_UP.V1_DN****
LEF1_UP.V1_UP****
EGFR_UP.V1_DN****
GCNP_SHH_UP_LATE.V1_DN***
LEF1_UP.V1_DN***
RB_P130_DN.V1_DN***
NFE2L2.V2***
Category
C1
C2
C3
C4
C5
C6
Group
High risk
Low risk
ssGSEA
0.5
0
−0.5
B
KEGG_UBIQUITIN_MEDIATED_PROTEOLYSIS
P = 5.50113589163462e−10
C
KEGG_FATTY_ACID_METABOLISM
P = 1.74589353839356e−08
D
KEGG_PEROXISOME
P = 3.26645656643532e−07
E
GOBP_POSITIVE_REGULATION_OF_HISTONE_H3_K27_METHYLATI
P = 7.77031665919557e−12
F
GOBP_REGULATION_OF_HISTONE_H3_K27_METHYLATION
P = 8.03476506852505e−11
G
GOBP_RESPONSE_TO_STAUROSPORINE
P = 9.66418610556898e−11
t−SNE1
t−SNE2
risk
high
low
PTMRS
H
corr
pearson's p
<0.01
<0.05
>=0.05
pearson's r
pos
neg
IFNG_signature
APM
Base_excision_repair
Cell_cycle
DNA_replication
Fanconi_anemia_pathway
Homologous_recombination
MicroRNAs_in_cancer
Mismatch_Repair
Nucleotide_excision_repair
Viral_carcinogenesis
Oocyte_meiosis
P53_signaling_pathway
Progesterone_mediated_oocyte_maturation
Proteasome
Pyrimidine_metabolism
Spliceosome
PTMRS
Step1
Step2
Step3
Step4.T cell.recruiting
Step4.CD4 T cell.recruiting
Step4.CD8 T cell.recruiting
Step4.Th1 cell.recruiting
Step4.Dendritic cell.recruiting
Step4.Th22 cell.recruiting
Step4.Macrophage.recruiting
Step4.Monocyte.recruiting
Step4.Neutrophil.recruiting
Step4.NK cell.recruiting
Step4.Eosinophil.recruiting
Step4.Basophil.recruiting
Step4.Th17 cell.recruiting
Step4.B cell.recruiting
Step4.Th2 cell.recruiting
Step4.Treg cell.recruiting
Step4.MDSC.recruiting
Step5
Step6
Step7
