# Supplementary material for: From multi-omics to functional validation: the PTMRS stratifies TME and positions PDGFRB in CRC biology
Source: Front Immunol. 2026 Jan 12;16:1728291. doi: 10.3389/fimmu.2025.1728291 (PMC12832729; doi:10.3389/fimmu.2025.1728291)
Supplement: Supplementary file 1 [file Supfig5.pdf › Supplementary data/Supfig5.pdf]

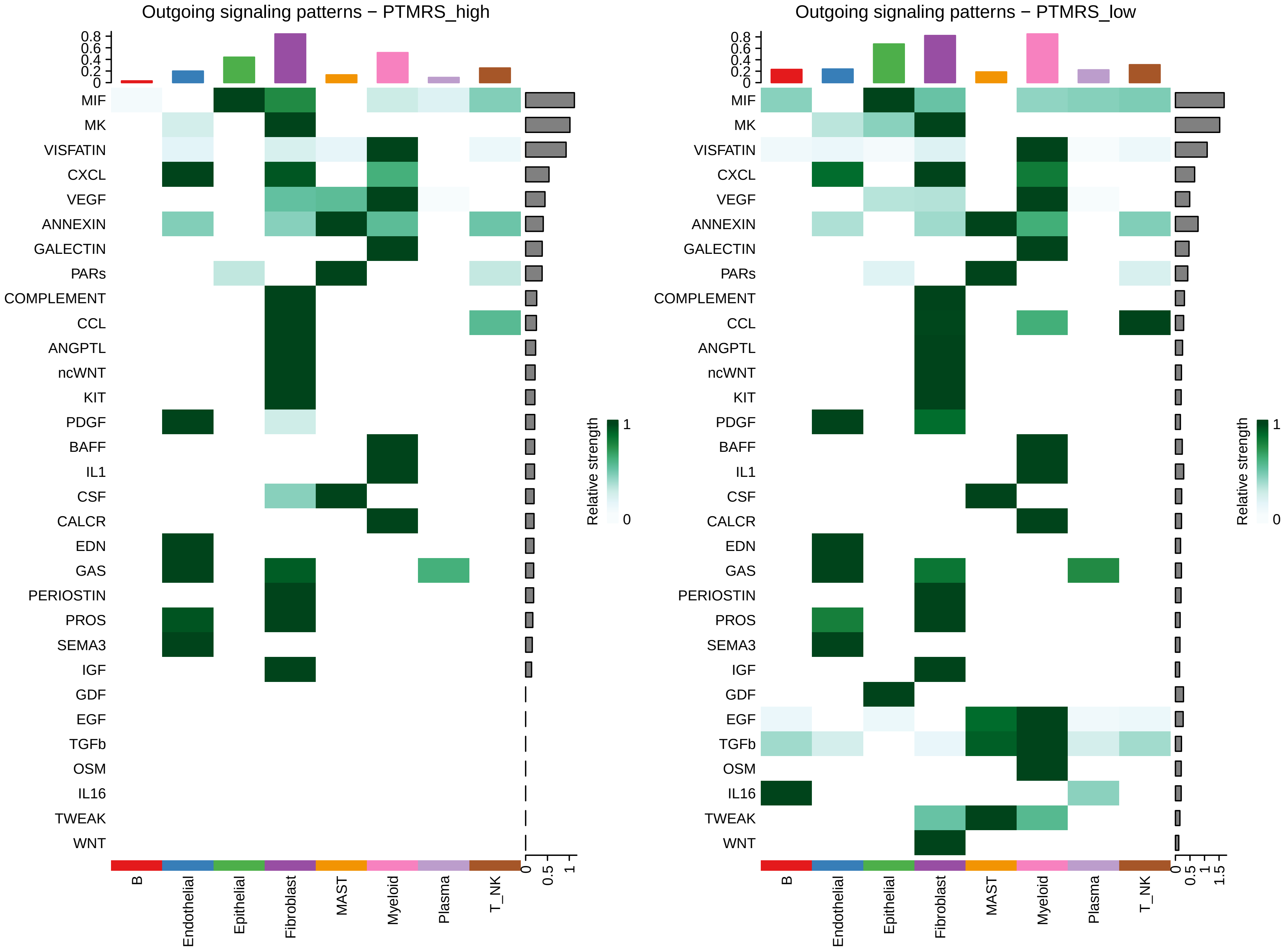

Outgoing signaling patterns − PTMRS_high
Outgoing signaling patterns − PTMRS_low
MIF
MK
VISFATIN
CXCL
VEGF
ANNEXIN
GALECTIN
PARs
COMPLEMENT
CCL
ANGPTL
ncWNT
KIT
PDGF
BAFF
IL1
CSF
CALCR
EDN
GAS
PERIOSTIN
PROS
SEMA3
IGF
GDF
EGF
TGFb
OSM
IL16
TWEAK
WNT
B
Endothelial
Epithelial
Fibroblast
MAST
Myeloid
Plasma
T_NK
Relative strength
0
1
0.8
0.6
0.4
0.2
0.5
1.5
